# Are Dogs Able to Communicate with Their Owners about a Desirable Food in a Referential and Intentional Way?

**DOI:** 10.1371/journal.pone.0108003

**Published:** 2014-09-18

**Authors:** Carine Savalli, César Ades, Florence Gaunet

**Affiliations:** 1 Department of Public Politics and Public Health, Federal University of São Paulo, Santos, SP, Brazil; 2 Department of Experimental Psychology, University of São Paulo, São Paulo, SP, Brazil; 3 Laboratoire de Psychologie Cognitive, UMR7290, Aix-Marseille Université/CNRS, Pôle 3C, Marseille, France; University of Lincoln, United Kingdom

## Abstract

The ability of dogs to use human communicative signals has been exhaustively studied. However, few studies have focused on the production of communicative signals by dogs. The current study investigated if dogs are able to communicate by using directional signals towards some desirable object in the environment and also if they show an apparent intention to manipulate their owner’s behavior in order to receive it. Some operational criteria were used to investigate referential and intentional communication: the signal should be influenced by the audience and by the recipient’s direction of visual attention; the sender should display gaze alternations between the recipient and the object and attention-getting behaviors, and, finally, the sender should persist and elaborate the communication when attempts to manipulate the recipient failed. Aiming to investigate these criteria in dogs, 29 subjects were tested using an experimental set up in which they could see a desirable but unreachable food and they needed the cooperation of their owners in order to receive it. This study found evidence of all operational criteria, especially for gaze alternation between the owner and the food, which suggested that some dogs’ communicative behaviors could be functionally referential and intentional. Nevertheless, similar to other studies about social cognition in animals, it is not possible to distinguish if the dog’s behaviors are based on simple mechanisms or on a theory of mind about their owners.

## Introduction

Communication with humans is a central feature of the social life of pet dogs. A number of studies have been done in order to assess the use of human deictic gestures by dogs and the role of experience in learning and shaping this ability [1]–[4]. However, only a few studies have investigated if dogs produce signals in order to communicate referentially, i.e. to show an object to a recipient, and intentionally, i.e. with the “goal” of manipulating the recipient’s behavior [5]–[7]. This is a crucial issue since most of the literature on this topic concern human infants and apes only [8]–[26]; other species have only been studied very recently [5]–[7], [27]–[30] and the present study is included in this category, evidencing the extent to which processes shown in human primates and apes are shared with other species.

In 12-month-old human infants the emergence of referentiality and intentionality is investigated prior to the development of language, when they start to use deictic gestures, such as designate a target with referential intentions and cooperative purposes as an attempt to establish joint attention [8]–[10].

Since communicative intents cannot be directly measured, Bates and colleagues [11] described some operational criteria that reveal the intentionality of signal production in preverbal infants, which were later used by Leavens and colleagues [12] in chimpanzees. These criteria, usually used to distinguish intentional from involuntary signal production, are: (a) the signal is used socially, i.e. an audience is required to exhibit the signal; (b) there are successive gaze alternations between the recipient and the object to be communicated; (c) the sender displays apparent attention-getting behaviors; (d) there is an influence of the recipient’s direction of attention; (e) there is persistence and (f) elaboration of communication when previous attempts to manipulate the recipient fail. By using this approach, the referentiality is associated to the presence and direction of gaze alternations and attention-getting behaviors (criteria b and c). According to Schel and colleagues [13] when these criteria are considered separately, they can be easily challenged by lower-level explanations as associative or emotional processes. For instance, the audience effect could be mediated by differing arousal levels caused by the presence of other individuals, and, sensitivity to the recipient’s direction of attention could be a learned discriminative response. In fact, what strengthens the explanation of intentionality for the signals is the combined evidence provided by multiple criteria.

Pointing or begging gestures in great apes have often been interpreted as a form of functionally referential and intentional communication [12], [14]–[16]. Apes use pointing and gaze alternation as referential signals [17], [18] and persist when their goal is not reached [12], [19]. These behaviors are influenced by the audience [20], [21] and by the direction of visual attention of a human observer [22]–[25]. Chimpanzees also present more multiple gestures when receiving an unwanted food instead of the desirable one [12]. A recent study also found that wild chimpanzees repeated gestures when a response of a conspecific reached partially their goal or substituted the original gesture if a response was incongruent, while they ceased communication when the recipient’s response was what they expected, which was interpreted as evidence of persistence and elaboration [19]. Moreover, chimpanzees [12] and orangutans [26] can distinguish between being completely or partially misunderstood (when an unwanted food or part of the desirable food is given, respectively). Compared to gestural research, intentionality has scarcely been addressed for ape’s vocalization. Recently, Schel and colleagues [13] found that vocalizations in wild chimpanzees when facing a potential threat, such as a snake, were not simply reflexive and unintentional, as an emotional response to their own risk, but socially produced and preceded by visual checking of the audience and gaze alternations. Additionally, individuals were more likely to persist in producing calls until all group members were safe from the predator.

Domestic dogs were also described manipulating human partners in order to reach food or toys, through signals, such as gaze alternation between a recipient and an object, which is considered to be one of the hallmarks of functionally referential and intentional communication [5]–[7], [27]. Hare and colleagues [4] presented the first evidence for communicative abilities in dogs. In one of their studies, two dogs witnessed a human hiding food and they were given the opportunity to lead another naive human to this food. One of the dogs was able to lead the naive person to the place where the food was hidden, even though its gazes and vocalizations did not differ according to human’s body orientations (facing the dog or back turned) or with eyes closed. During a short period of time, when the naive person was not present in the experimental setting, this dog waited sitting, which indicates that those signals were socially used. More recently Kaminski and colleagues [28] presented evidence that dogs communicate to request what they want, but not to inform the experimenter about an object that was not of their interest. However, the authors stated that the dogs were more motivated to inform when the owners took the role of the experimenter; which was evaluated using the human’s performance in finding the object based on the dogs’ behaviors. It should be highlighted that the results observed in this study [28] are based on the human’s ability to interpret the dog’s behavior. Therefore, further investigations regarding dogs’ communicative signals would be informative in this matter.

It has been observed that dogs direct their owner’s attention towards the location of a desirable hidden or visible target with gaze alternations and with their own position, as a local enhancement signal, in a functionally referential way [5], [6], [27], [31]. Dogs also prefer to choose an attentive person instead of an inattentive one to beg for food [32], [33] and catch less food from the floor when the human is looking at them than when the human is inattentive [34]. Moreover, dogs are sensitive to human’s visual perspective [35], [36]: in the presence of an opaque barrier blocking the human’s view of a forbidden food, dogs took more of this forbidden food [35]. The influence of humans' direction of attention on dogs’ communicative signals has been observed when facing a new and potentially scary object [37] and in an unsolvable task [38], but it has not been addressed when dogs can beg for food. Finally, one study investigated the persistence and elaboration of communication in dogs [7]: dogs showed persistence when an unfamiliar object was returned instead of a desirable toy, but no new behaviors were observed after receiving the unfamiliar object. Such response was interpreted as an absence of elaboration in communication.

Rossi and Ades [39] trained a mongrel dog, Sofia, to communicate her desires such as *food*, *water*, *crate*, *walk*, *toy* and *petting*, by pressing her paw on a keyboard with arbitrary signs (lexigrams). She used the keyboard in the presence of a human, and was influenced by the human’s visual access to it [40]. Moreover, she persisted when she did not obtain a response, which means that some aspects of intentionality are present in this special form of communication.

According to the aforementioned studies, the criteria described by Bates and colleagues [11] have been addressed separately in different dog samples. However, if all operational criteria are essential to characterize the communication as referential and intentional, an additional study that evaluates all of them at the same time in the same sample is required. Therefore, the aim of the present study was to investigate all operational criteria (a-f) in a single dog sample by means of a combination of experiments that simulated a naturalistic situation in which there was a visible but inaccessible food in two possible locations; dogs needed to communicate with their owners in order to get it. We manipulated the presence of the owner and food in the room as well as the direction of the owner’s attention and the outcome after a period in which the dogs could communicate about the food. Dogs’ behavior, combined with their position in the room, were analyzed when: (i) only food was present in the room (absence of owner condition); (ii) only the owner was present in the room (absence of food condition); (iii) both owner and food were in the room and the owner had his/her back turned (owner turned condition); both owner and food were in the room and after communicating the dog received (iv) the entire food (success in communication), (v) half of the food (partial failure in communication), or (vi) an undesirable food (complete failure in communication). Suitable comparisons among these six experimental conditions allowed an extension of previous studies [4]–[7] and considerations of all operational criteria for referentiality and intentionality (a–f) at the same time in a food begging context.

The current study differs from previous studies in dogs [5]–[7] regarding the procedures used and presented a number of benefits. The investigation of all criteria in a single sample can provide with stronger results since it allows the control of individual variation. The use of two possible locations also allowed us to evaluate if dogs use a directional component towards the food. When there is only one possible location for the target, as in Gaunet and Deputte [6], it is difficult to assure that those directional behaviors refer, in fact, to the target instead of any other information in that location. Moreover, the use of a visible target was intended to maximize the display of communicative behaviors, since Gaunet and Deputte [6] observed that dogs used less referential communicative behaviors when a desirable toy was hidden behind a door. A hidden food item or toy, as used in previous studies [5]–[7], may require working memory ability, and, even though some studies have already shown that dogs possess a certain level of this cognitive processing [41], [42], it is still not clear how it could interfere in the display of communicative signals (although a recent study [27] found no difference in the display of gaze alternations for visible and invisible target). More importantly, to the best of the authors’ knowledge the current study addressed for the first time the effect of the owner’s body direction on the production of communicative signals by dogs, especially gaze alternations, in a food begging situation. Finally, the benefit of choosing food instead of a toy as a target was that it could be easily divided into two pieces in the dog’s view, allowing the analysis of the effect of a partial failure in communication (when they received only half of the food).

Firstly, if dogs produced behavior towards the food with the goal of communicating with their owners (rather than just simply try to reach the food) we expected to find that they used these behaviors socially: more behaviors towards the food would be displayed when both owners and food were present in the room than when the owner was absent (criterion a). This is the first criterion to be validated since all other criteria require a social audience. Secondly, if dogs’ behaviors towards the food were referential, we expected that dogs would alternate gazes between the owner and the food and that they would display more communicative behaviors when both owner and food were present in the room than when the food was absent. Additionally, behaviors would divert towards the direction of the door through which a helper left with the food in the absence of food condition (criteria b and c) [5]–[7]. Thirdly, if dogs were sensitive to the direction of owners’ visual attention, they would modulate their visual and sonorous communicative behaviors in order to increase the odds of getting a response, presenting more visual behaviors towards the food when the owners were facing forwards and could see the food than when they had their back turned (criterion d). Fourthly, if the criterion of persistence was met, we expected that dogs would continue to exhibit communicative behaviors after either receiving half of the food (partial failure in communication) or the undesirable food (complete failure in communication), while they would display less or cease to exhibit these behaviors after receiving the entire food (success in communication) (criterion e) [7]. Finally, if dogs exhibit elaboration in communication, then multiple and alternative behaviors would also be more marked after either receiving half of the food or the undesirable food than after receiving the entire food (criterion f). The integration of behavior and location analyses intended to investigate if dogs would combine communicative behaviors with the use of their own position in the room in a functionally referential and intentional way.

## General Methods

### Subjects

Thirteen male and sixteen female adult pet dogs of different breeds took part in the study (mean age: 5.81±3.25 years old). According to their owners, the selected dogs usually displayed begging behaviors in the presence of food, and did not usually present signs of distress in unfamiliar places or in the absence of their owners. Owners also provided with information about dogs’ favorite food and were instructed to feed their dogs 5 to 6 hours before the experiment. Each dog was tested with its favorite food.

### Ethics Statement

This study was approved by the Committee for Ethical Research in Animals (CEPA) of the Institute of Psychology of USP (University of São Paulo) (process number 004.2012). Owners gave consent for their dogs’ participation in this study.

### Experimental settings

**Figure 1:**
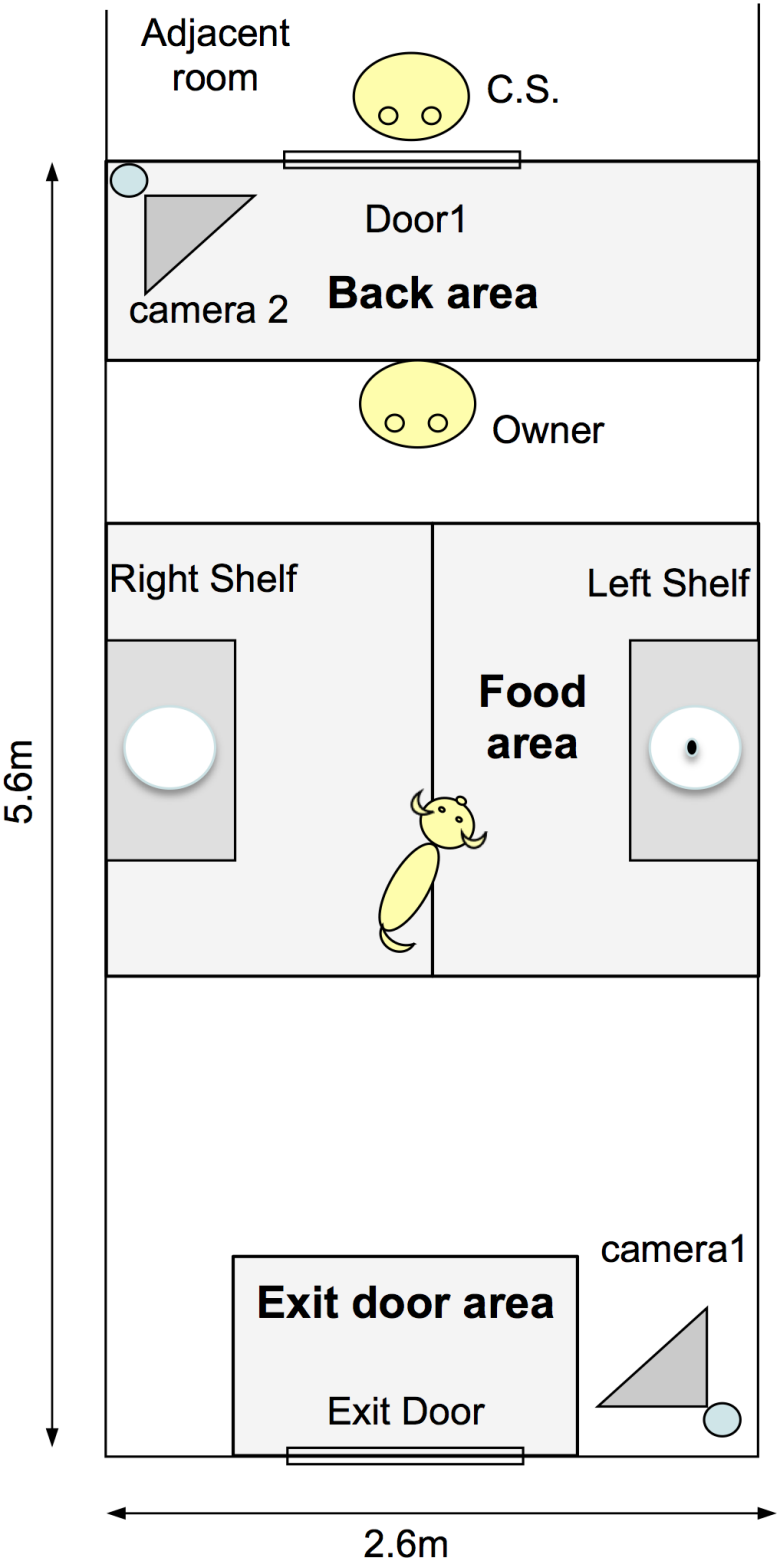
Experimental setting.

**Figure 2:**
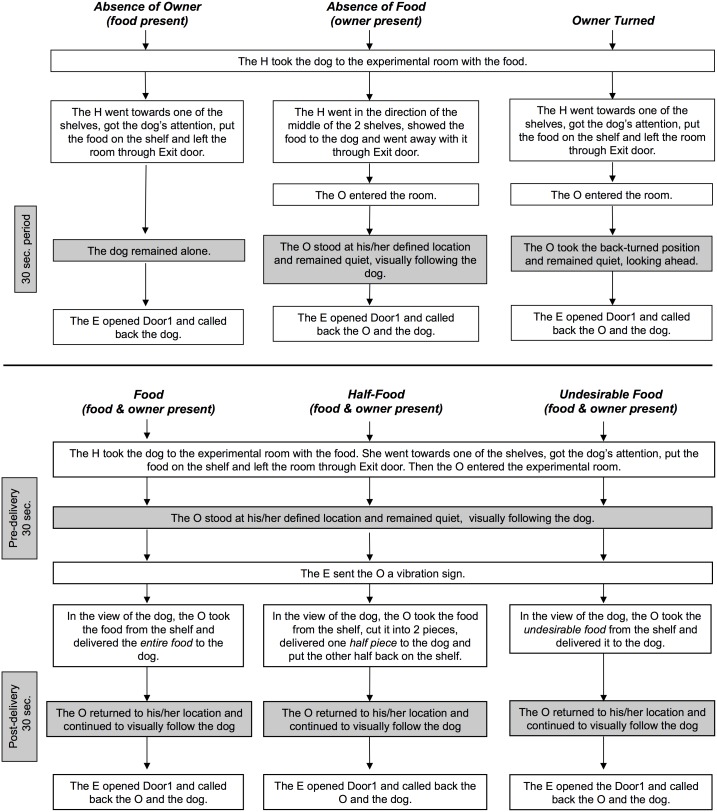
Sequence of actions by the owner (O), helper (H) and experimenter (E) for each condition.

In the experimental room (Figure 1) there were two shelves: in one of them (named food shelf), the dog’s favorite food was placed, by alternating randomly across trials for each dog. The shelves could be positioned at two possible heights to make the food unreachable for dogs of different sizes. The dogs could see the food and put the paw on the shelves if they stretched their bodies, but the food still remained unreachable. There was a unidirectional microphone placed in the center of the room and attached to a crossbar connecting the two sides of the room. The owner carried a vibrator collar that was remotely activated by the experimenter (C.S.) to signal specific moments during some trials (described in the procedures in Figure 2) as covertly as possible in order to avoid attracting the dog’s attention. Two cameras recorded all trials. A helper (F.T.)’s task was to bring the dog and the food into the room and then leave the room through the exit door.

### Familiarization phase

The owner stood at the marked location (see Figure 1) and the helper stood in front of the owner on the other side of the room. The helper put the food on one of the shelves and returned to her place. Then the owner immediately called the dog by its name, went towards that shelf, got the food and gave it to the dog. This procedure was repeated, with alternation between the shelves, until the dog looked at the owner right after the helper had placed the food on the shelf (average number of repetitions required to reach the criterion: 11.2±3.9 times, ranging from 6 to 21).

### Experimental phase

Prior to the tests, a brief explanation of the experimental conditions was given to the owner. The experiment consisted of six conditions presented sequentially once to each dog; the order of these conditions was counterbalanced across dogs, using a block randomization, as well as the side on which the food was positioned (left or right), with the restriction that one side could not be used more than twice consecutively.

The experimenter remained in the adjacent room, controlling the entries of the helper, owner and dog into the experimental room. The conditions were (see Figure 2 for procedural details):

*-* Absence of Food: a 30-second trial during which only owner and dog were present.
*-* Absence of Owner: a 30-second trial during which only food and dog were present.
*-* Owner Turned: a 30-second trial during which both owner and food were present, and the owner had his/her back turned to the food.
*-* Food (success in communication): a 30-second trial during which both owner and food were present (pre-delivery phase) followed by a 30-second trial after the owner delivered the entire food to the dog (post-delivery phase). Please see Video S1 for a demonstration.
*-* Half-Food (partial failure in communication): a 30-second trial during which both owner and food were present (pre-delivery phase) followed by a 30-second trial after the owner delivered only half of the food to the dog, and returned the other half to the shelf (post-delivery phase). Please see Video S2 for a demonstration.
*-* Undesirable Food (complete failure in communication): a 30-second trial during which both owner and food were present (pre-delivery phase) followed by a 30-second trial after the owner delivered an undesirable food (placed behind the food before the dog entered the room) to the dog, while the desirable food was left on the shelf (post-delivery phase).

The undesirable food was chosen before testing. Some vegetables were offered to the dog and the most unwanted one, according to the experimenter and owner’s perceptions, was used in the Undesirable Food condition, usually scarlet eggplant or okra.

The testing included intervals of approximately five minutes between conditions. Dogs received a piece of food from the owner at the end of each condition in a spontaneous manner, so that they could associate the situation with availability of food. This did not result in learning across conditions (see Results for confirmation).

### Behavioral analysis and coding

We collected data regarding multimodal (visual and acoustic) behaviors and locations of the dogs with Actogram Kronos software (Octarés Edition). For each dog, we defined overlapping behaviors (i.e. not mutually exclusive), with or without movements [4]–[7], [31]:

- *Gaze Owner*: the dog’s head/nose was oriented towards the owner's head face;
- *Gaze Food*: the dog’s head/nose was oriented towards the food;
- *Gaze Alternation between the owner and the food*: this consisted of a gaze at the owner’s face followed by a gaze at the food (or vice-versa) [12];
- *Gaze Exit door*: the dog’s head/nose was oriented towards the exit door;
- *Gaze Alternation between the owner and the exit door*: this consisted of a gaze at the owner’s face followed by a gaze at the exit door (or vice-versa);
- *Gaze Shelves*: the dog’s head/nose was oriented towards one or other of the empty shelves. It was coded for the Absence of Food condition only. It was calculated by averaging the relative durations or frequencies of gazes at the left and right empty shelf for each dog.
- *Gaze Alternation between the owner and the shelves*: this consisted of a gaze at the owner’s face followed by a gaze at one or other of the empty shelves (or vice-versa). It was coded for the Absence of Food condition only. It was calculated by averaging the frequencies of gaze alternations between the owner and the left empty shelf, and, between the owner and the right empty shelf for each dog.
- *Gaze Owner’s back*: the dog’s head/nose was oriented towards the owner's back. It was coded for the Owner Turned condition only;
- *Gaze Alternation between the owner’s back and the food*: this consisted of a gaze at the owner’s back followed directly by a gaze at the food (or vice-versa). It was coded for the Owner Turned condition only;
- *Vocalization*: the dog barked and/or whined;
- *Silent Mouth Licking:* the dog displayed non-sonorous (silent) mouth licking;
- *Sonorous Mouth Licking*: the dog displayed sonorous (noisy) mouth licking that was audible on the video recording;
- *Contact Owner*: the dog touched the owner with any part of its body.

We used the total duration (30 seconds) of each trial to calculate the relative durations and frequencies of all behaviors, except for gaze alternation variables (absolute frequencies only). Since behaviors could overlap (e.g. Gaze Owner and Vocalization at the same time), the duration and frequency of each behavior were calculated by considering all occasions on which it appeared, regardless of whether it was alone or combined with any other behavior.

For the study of elaboration, multiple behaviors were defined as the combination of Gaze Owner or Gaze Food with at least one additional behavior such as Contact Owner, Vocalization, Mouth Licking (silent or sonorous) and:

- *Sniff Food*: the dog sniffed the food;
- *Paw Food*: the dog put a paw on the food shelf;
- *Point Food with Muzzle*: the dog put a paw on the shelf with its muzzle oriented toward the food.

For both pre and post-delivery phases of Food, Half-Food and Undesirable Food conditions, the dogs were dichotomously classified as having either exhibited multiple behaviors or not and we calculated the proportion of dogs that exhibited multiple behaviors. The absolute frequency of multiple behaviors for each dog was also analyzed. Since right after eating food in the post-delivery phases of Food or Half-Food conditions, mouth lickings (silent or sonorous) could be just a mouth cleaning reaction rather than a communicative behavior, analyses regarding the persistence and elaboration criteria took into account only the occurrences of mouth lickings that happened after 10 seconds the dogs had eaten the food.

Moreover, the dogs were dichotomously classified as having either exhibited alternative behaviors or not (behaviors exhibited during post-delivery phase that were not displayed during pre-delivery phase) and for the three conditions we calculated the proportion of dogs that exhibited alternative behaviors.

### Location analysis and coding

In order to evaluate if the dogs used their own location as a local enhancement signal [6], we computed the time the subjects spent (duration only) in mutually exclusive areas, using the location of their two front legs. For the coding, we used a transparent mask, with the areas marked, which was placed onto the computer screen. The areas coded were (see Figure 1):

- *Food area*: when the dog was in the 1.2 m×1.8 m rectangle closest to the food shelf;
- *Exit door area*: when the dog was in the 0.8 m×1.1 m rectangle closest to the exit door;
- *Shelves area*: when the dog was in one of the two areas adjacent to the shelves (two rectangles of 1.2 m×1.8 m). It was coded for Absence of Food only. It was calculated by averaging the durations in the left and the right areas;
- *Back area*: when the dog was in the 2.6 m×0.8 m rectangle behind the position of the owner.

We used the total duration (30 seconds) of each trial to calculate the relative duration spent in each area.

To make surfaces comparable (e.g. *Food area* with *Exit door area*, or, *Food area* with *Back area*), a correction that represented an index of proportionality of surfaces was applied.

### Location and behavior combined analysis

We additionally computed the relative durations (only) of certain combinations of locations and behaviors and absolute frequencies when the combination involved gaze alternation:

- *Food area* combined with: *Gaze Owner, Gaze Food, Gaze Alternation between the owner and the food, Sonorous Mouth Licking.* For the Owner Turned condition we also analyzed the combination of *Food area* with *Gaze owner’s back* and *Gaze Alternation between the owner’s back and the food*;
- *Exit door area* combined with: *Gaze Exit door* and *Gaze Alternation between the owner and the exit door*;
- *Shelves area* combined with: *Gaze Owner, Sonorous Mouth Licking, Gaze Shelves* and *Gaze Alternation between the owner and the shelves* for the Absence of Food condition only;
- *Back area* combined with: *Gaze Owner, Gaze Food* and *Sonorous Mouth Licking* for the Owner Turned condition only.

In order to simplify the text, we refer below to relative or absolute frequencies and durations of variables as “frequencies” and “durations” respectively.

### Comparisons and statistical analyses

#### Control for the learning effect

In order to evaluate if there was a learning effect across conditions, the first and the last pre-delivery phases (from the Food, Half-Food or Undesirable Food conditions), according to the order of presentation for each dog, were compared for 4 relevant behaviors (durations of Gaze Owner and Gaze Food, time spent in the Food area, and frequency of Gaze Alternations between the owner and the food), using a two-sample Wilcoxon Signed-rank test.

#### Comparisons between the pre-delivery phases of Food, Half-Food and Undesirable Food conditions

**Figure 3:**
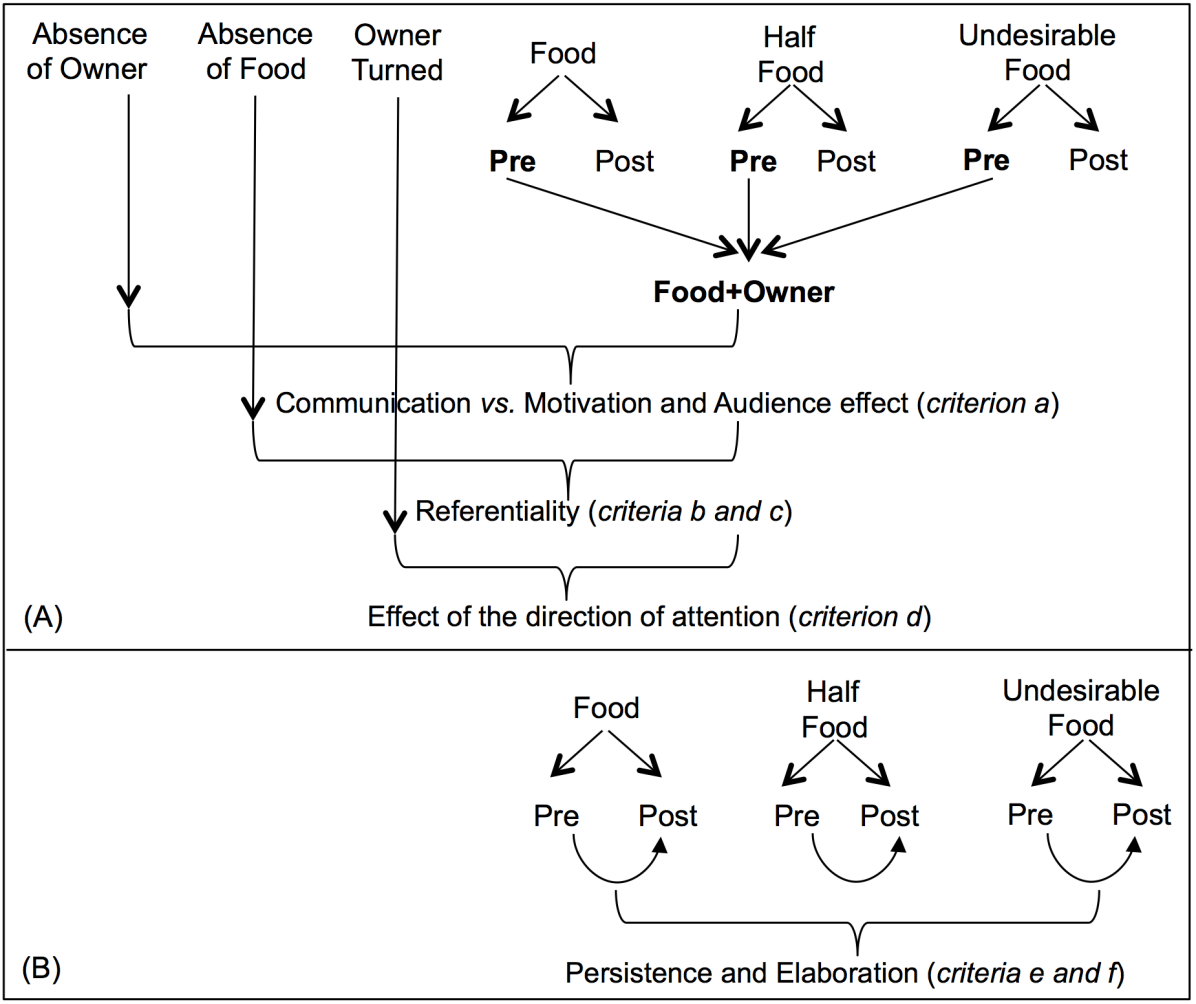
Comparisons between conditions and phases to test all criteria of referentiality and intentionality.

To control the stability of the experimental manipulations, the pre-delivery phases for Food, Half-Food and Undesirable Food conditions (that were procedurally identical and constituted the situation during which both food and owner were present in the room) were compared using a Friedman’s test for the following variables: durations and frequencies of Gaze Owner, Gaze Food, Vocalization, sonorous or silent Mouth Licking and Contact Owner, frequency of Gaze Alternations between the owner and the food, and the time spent in the Food area. Since no differences were found (see Results section) the data from the pre-delivery phases of these three conditions were pooled and called “Food+Owner” (see Figure 3A).

#### Selection of relevant variables for analyses

The durations and frequencies of the variables were first compared to zero in the Food+Owner condition with a one-sample Wilcoxon Signed-rank test. If in the presence of both food and owner (when the higher rate of communicative behaviors was expected) a given behavior or location (or a combination of them) rarely occurred, i.e., not significantly different from zero in duration or frequency, then it was not included in further analyses. Therefore, only variables significantly different from zero in this condition were evaluated regarding referentiality and intentionality.

#### Test of social use (criterion a): Comparisons between the Food+Owner and Absence of Owner conditions

As these two conditions differ according to the presence of the owner, this comparison, performed using the two-sample Wilcoxon Signed-rank tests, intended to evaluate the audience effect (see Figure 3A). If in the presence of both food and owner, a given behavior or location (or a combination of them) occurred more than in the absence of the owner, this behavior was considered communicative, since it was influenced by the audience.

Some behaviors could not occur in the absence of the owner (Gaze Owner, Gaze Alternations between the owner and the food and Contact Owner). Thus, they were considered communicative if they occurred frequently when both food and owner were present in the room (duration or frequency different from zero in Food+Owner).

#### Test of referentiality (criteria b and c): Comparisons between the Food+Owner and Absence of Food conditions

**Figure 4:**
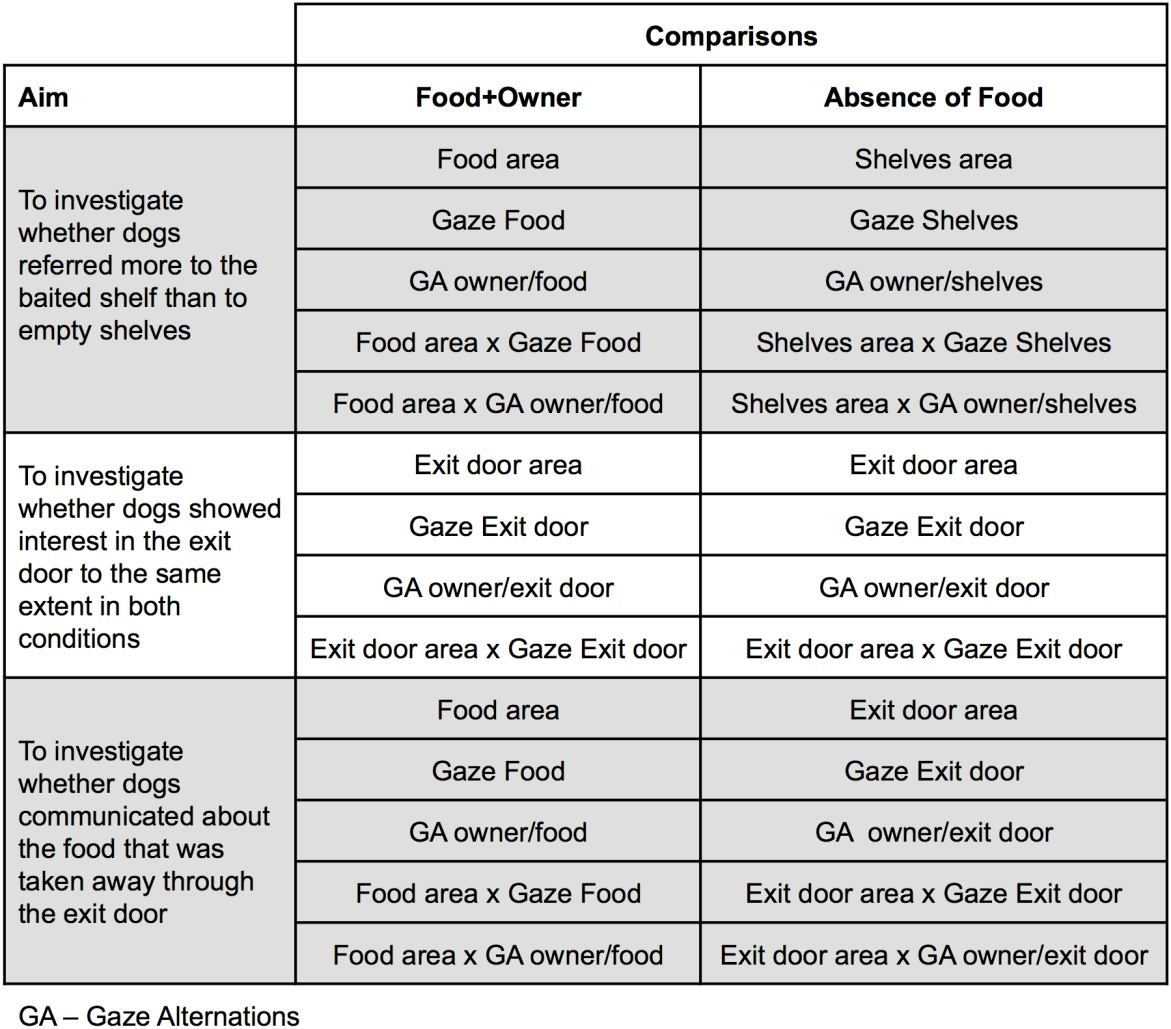
Additional comparisons to investigate the referentiality criterion.

As these two conditions differ according to the presence of the food, this comparison, performed using the two-sample Wilcoxon Signed-rank test, intended to evaluate the referentiality of dogs’ behaviors towards the food, especially the gaze alternation (see Figure 3A). A given behavior or location (or a combination of them) was considered functionally referential if it occurred more in the presence of both food and owner than in the absence of the food. Comparisons involving behaviors or locations (or combinations of them) towards the Exit door and the shelves in the Absence of Food condition (described in Figure 4) also provided with information about referentiality, since they revealed the directional component of behaviors towards the food.

#### Test of the owner’s direction of attention (criterion d): Comparisons between the Food+Owner and Owner Turned conditions

**Figure 5:**
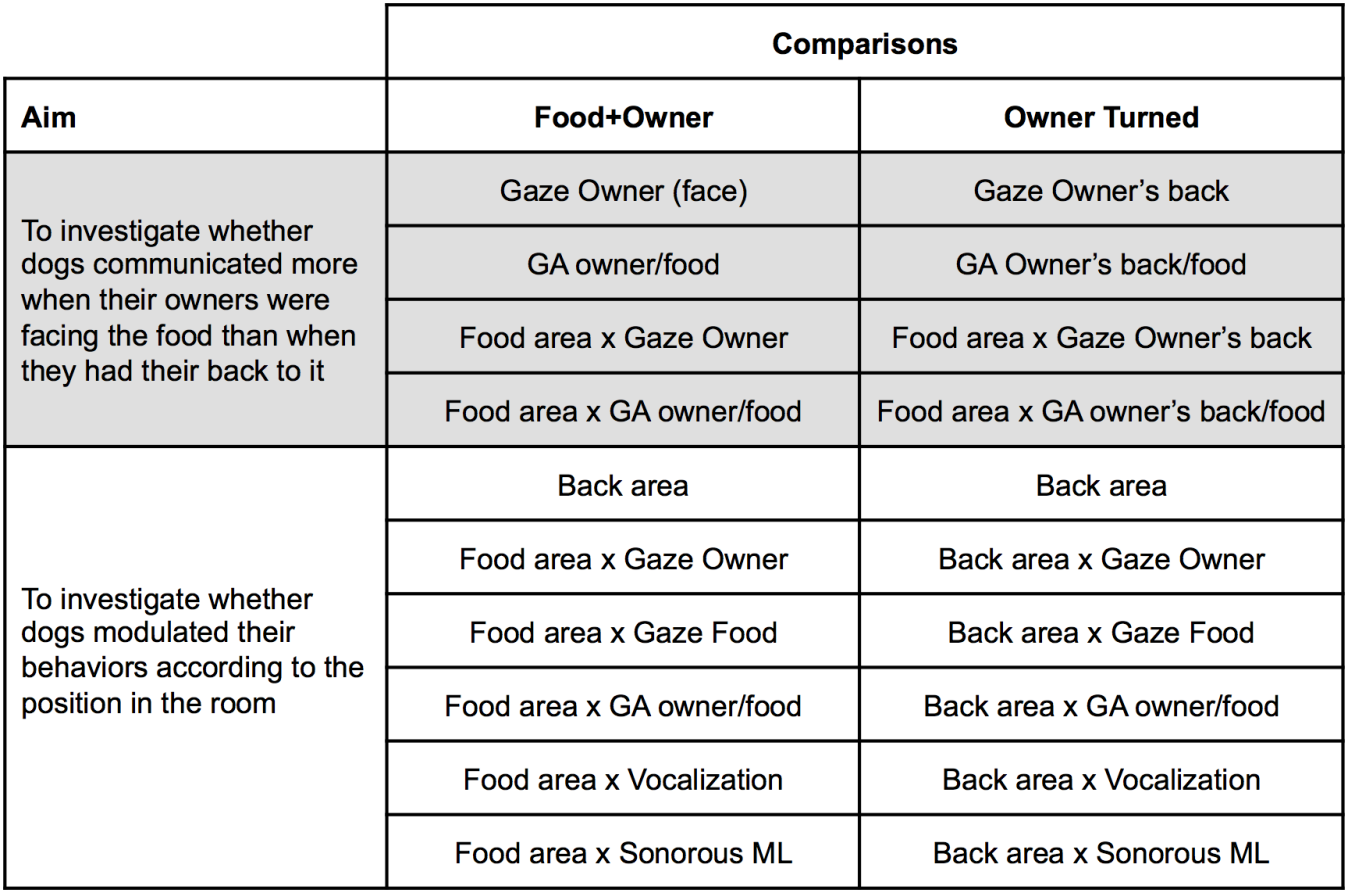
Additional comparisons to investigate the effect of the owner’s direction of attention.

As these two conditions differ according to the orientation of the owner’s body (facing or not facing the food), this comparison, performed using the two-sample Wilcoxon Signed-rank test, intended to evaluate the effect of the owner’s direction of attention (see Figure 3A). Evidence of this effect was considered to have been provided if a given behavior happened more when the owners were facing the food than when they had their back turned. Although duration and frequency of Vocalizations were not significantly different from zero in the Food+Owner condition (see Results), this sonorous behavior could have increased in the Owner Turned condition as a way to get the owner’s attention. Therefore, to test this hypothesis, we compared these two conditions regarding these variables. Comparisons involving gazing at the owners’ back in Owner Turned condition and modulation of behaviors regarding the locations Food area and Back area (described in Figure 5) also provided with information about this criterion.

#### Test of persistence and elaboration (criteria e and f): Comparisons between the pre and post-delivery phases of Food, Half-Food and Undesirable Food conditions

To evaluate persistence (criterion e), we performed comparisons between the pre vs. post-delivery phases for the Food, Half-Food and Undesirable Food conditions using the two-sample Wilcoxon Signed-rank test (Figure 3B). It was considered evidence of persistence if a given behavior or location (or combination of them) decreased after receiving the entire food (success in communication), but it did not decrease after receiving either half of the food (partial failure in communication) or the undesirable food (complete failure in communication). Additional comparisons between the post-delivery phase of Food with the post-delivery phase of the other two conditions were performed in order to confirm tendencies observed for the pre vs. post comparisons.

These comparisons also addressed elaboration (criterion f) (Figure 3B). In order to compare the proportion of dogs that exhibited multiple behaviors across the three pre-delivery phases in the Food, Half-Food and Undesirable Food conditions, Cochran’s Q test was applied (in order to confirm the stability of the experimental manipulations in the three pre-delivery phases). Then, the pre *vs.* post-delivery comparisons were performed using McNemar’s test. Regarding the absolute frequency of multiple behaviors, the three pre-delivery phases were compared using a Friedman’s test (in order to confirm the stability of the experimental manipulations in the three pre-delivery phases) and the pre *vs.* post-delivery comparisons were performed using two-sample Wilcoxon Signed-rank test. It was considered evidence of elaboration if multiple behaviors decreased after receiving the entire food, but did not decrease after receiving either half of the food or the undesirable food. Comparisons between the Food condition with the other two conditions in the post-delivery phase were performed in order to confirm tendencies observed for the pre *vs.* post comparisons. Finally, to compare the proportion of dogs that exhibited alternative behaviors during the post-delivery phase that were not displayed during the pre-delivery phase across the three conditions Food, Half-Food and Undesirable Food, Cochran’s Q test was applied. The multiple and alternative behaviors that occurred only in the Food area were also analyzed.

Correction for multiple comparisons were only necessary for persistence and elaboration analyses since for each dependent variable the three conditions (Food, Half-Food and Undesirable Food) were compared by means of 5 contrasts (3 pre *vs.* post comparisons and 2 comparisons in post delivery-phases -i.e., Food *vs.* Half-Food and Food *vs.* Undesirable Food). A false discovery rate correction was adopted (FDR BL adjustment) for correction for the use of multiple comparisons [6], [43], [44].

We used SAS software 9.2, SAS Institute Inc., Cary, NC, USA for all statistical analyses and a 5% significance level was applied. All tests were two-tailed.

A second naive observer independently coded 34% of the sample (chosen randomly) and the Kendall’s concordance coefficient was calculated. For the Food condition (pre and post-delivery phases) the inter-observer agreement was assessed for the following variables: the duration of Gaze Owner (*W* = 0.988), Gaze Food (*W* = 0.988), Gaze Exit door (*W* = 0.995), combination of Gaze Food with Vocalization (*W* = 1), with Sonorous Mouth Licking (*W* = 1) and with Paw Food and Sniff Food (*W* = 1), the number of Gaze Alternation between the owner and the food (*W* = 0.998) and the time spent in the Food area (*W* = 0.995) and Exit door area (*W* = 1). For the Absence of Food condition the inter-observer agreement was assessed for the Gaze Alternation between the owner and the exit door (*W* = 1). Results indicated a good agreement between raters.

In the figures, data are represented using *boxplots*, whiskers extend to the smallest and largest values and outliers have been excluded.

## Results

No learning effect was found for the variables tested (see statistics in Table S1). Additionally, the three pre-delivery phases of Food, Half-Food and Undesirable Food conditions did not differ for all variables analyzed (see statistics in Table S2). The data from these pre-delivery phases were pooled by dog and by variable (using the median of the 3 values) and named “Food+Owner” condition.

Durations and frequencies of Gaze Owner, Gaze Food and Sonorous Mouth Licking, the frequency of Gaze Alternations between the owner and the food, the time spent in Food area, as well as the combination of Food area with Gaze Owner, Gaze Food and Gaze Alternation between the owner and the food occurred frequently during the Food+Owner condition (significantly different from zero: see statistics Table S3). On the other hand, the durations and frequencies of Vocalization, Silent Mouth Licking and Contact Owner did not differ from zero. The combinations of Food area with the two acoustic behaviors (Vocalization and Sonorous Mouth Licking) were also tested and they also did not differ from zero (see statistics in Table S3). Further analyses were not performed for these variables because they rarely occurred during the Food+Owner condition, except for Vocalization that was analyzed for Owner Turned since it is an acoustic behavior and could increase in this condition.

### Tests of criteria a–f of referentiality and intentionality

#### Test of social use (criterion a): Comparisons between the Food+Owner and Absence of Owner conditions

**Figure 6:**
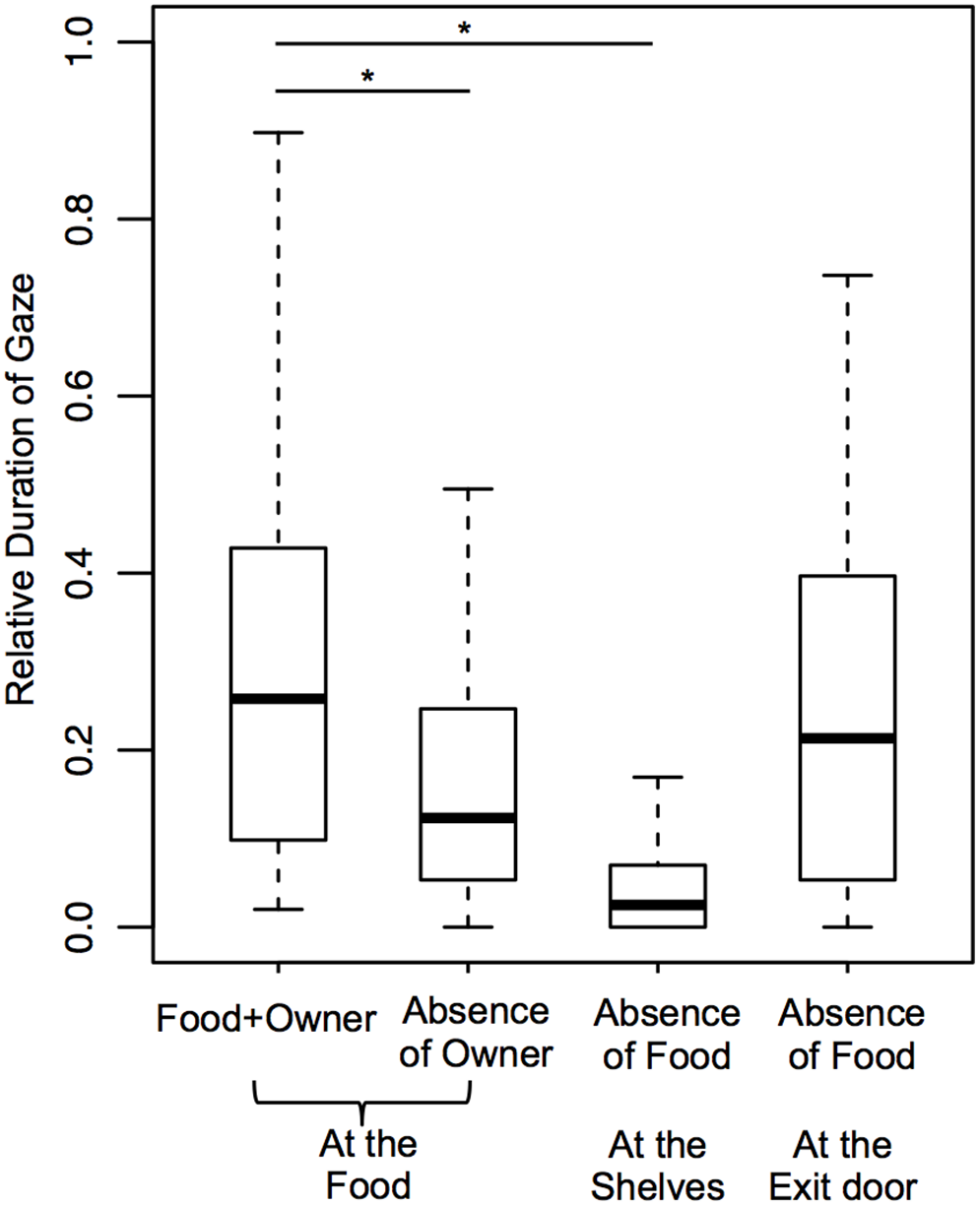
Relative duration of Gaze Food during Food+Owner and Absence of Owner, and, relative duration of Gaze Shelves and Gaze Exit door during Absence of Food.

The durations of Gaze Food (Figure 6), Sonorous Mouth Licking and the time spent in Food area were significantly greater during Food+Owner than during Absence of Owner. However, no difference was found between these two conditions for the frequencies of Gaze Food and Sonorous Mouth Licking, as well as for the time spent in Food area combined with Gaze Food (see statistics in Table 1). For behaviors that involved the owner (Gaze Owner and Gaze alternation between the owner and the food) it was not possible to perform this comparison, however since they occurred frequently during the Food+Owner condition, they were considered to be communicative.

**Table 1:** Medians (Interquartile ranges-IQR) for the variables and two-sample Wilcoxon Signed-rank tests for comparisons regarding the audience effect. Significant differences are in bold.

	Duration: Median (IQR)				Frequency: Median (IQR)			
Variables	Food+Owner	Absence of Owner	*T*	*p*	Food+Owner	Absence of Owner	*T*	*p*
Gaze food	0.26 (0.33)	0.12 (0.20)	95.5	**0.037**	0.13 (0.10)	0.10 (0.13)	9	0.803
Sonorous Mouth Licking	0 (0)	0 (0)	13	**0.031**	0 (0)	0 (0)	10.5	0.109
Food area	0.48 (0.68)	0.16 (0.28)	107	**0.012**	–			
Food area×Gaze Food	0.13 (0.33)	0.07 (0.18)	32	0.452	–			

#### Test of referentiality (criteria b and c): Comparisons between the Food+Owner and Absence of Food conditions

The duration of Gaze Owner during Food+Owner was greater than during Absence of Food (T = 92.5, p = 0.043), also when Gaze Owner combined with Food area during Food+Owner was compared with Gaze Owner combined with Shelves area during Absence of Food (duration) (T = 92, p = 0.034). No difference was found between these two conditions regarding the frequency of Gaze Owner (T = 69, p = 0.098). There was no effect of the absence of the food on the duration (T = 6, p = 0.520) and frequency (T = 0.5, p = 1.000) of Sonorous Mouth Licking.

**Figure 7:**
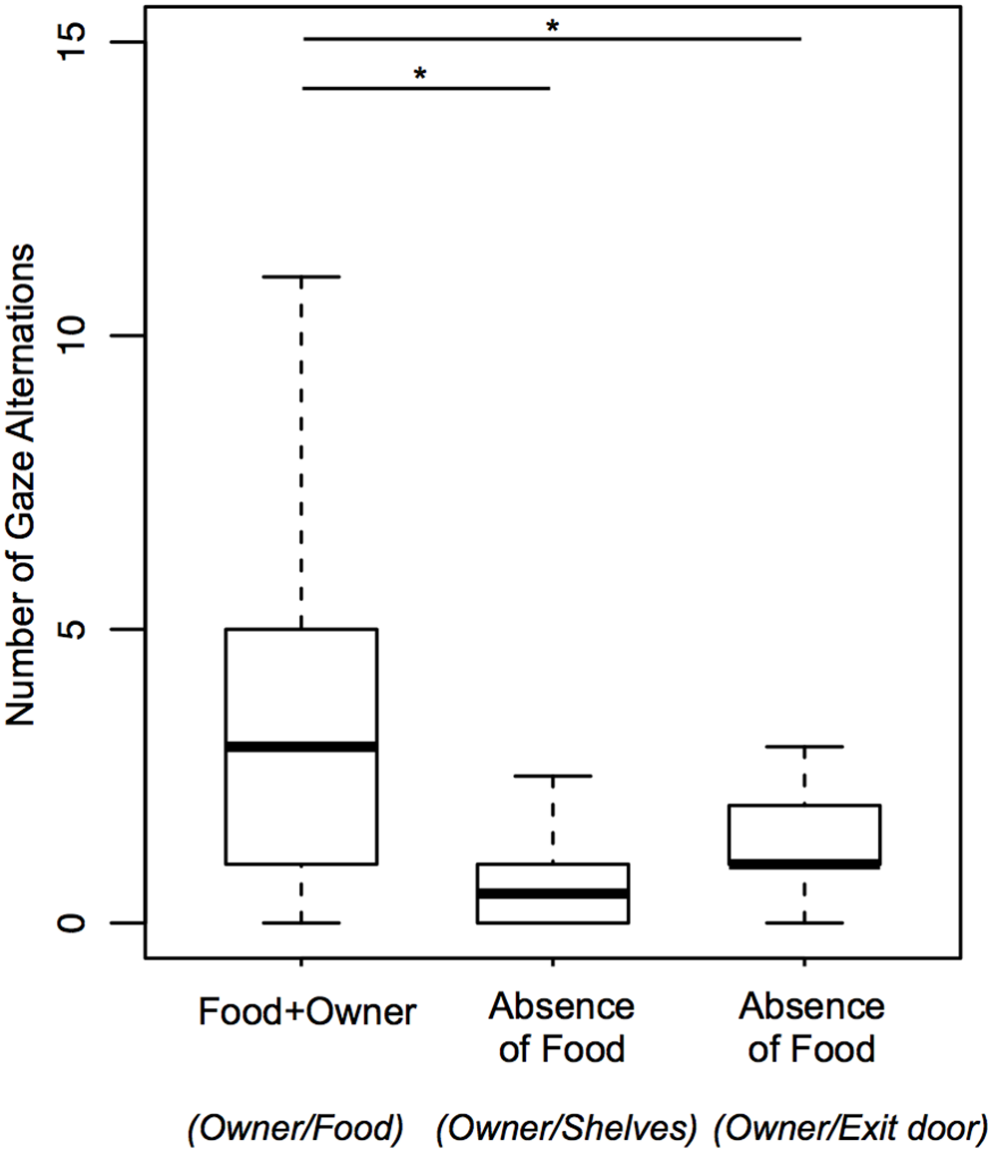
Frequencies of gaze alternation: between the owner and the food during Food+Owner; between the owner and the shelves, and, between the owner and Exit door during Absence of Food.

Comparisons of behaviors towards the food during the Food+Owner condition with behaviors towards the shelves during the Absence of Food condition intended to evaluate if the dogs indicated more the food shelf than the empty shelves (see statistics in Table 2). The time spent in Food area during Food+Owner was greater than that the time spent in Shelves area during Absence of Food. The duration and frequency of Gaze Food and the frequency of Gaze Alternations between the owner and the food during Food+Owner were significantly greater than those towards the shelves during Absence of Food (Figure 6 and 7), as well as when these behaviors were analyzed only in Food area and Shelves area (duration), respectively for each condition. Therefore, the dogs tended to indicate more the food shelf than the empty shelves.

**Table 2:** Medians (Interquartile ranges-IQR) for the variables and two-sample Wilcoxon Signed-rank tests for comparisons regarding referentiality. Significant differences are in bold.

Comparisons		Duration: Median (IQR)				Frequency: Median (IQR)			
Food+Owner variable	Absence of Food variable	Food+Owner	Absence of Food	*T*	*p*	Food+Owner	Absence of Food	*T*	*p*
***Food (Food+Owner) vs. Shelves (Absence of Food)***									
Gaze Food	Gaze Shelves	0.26 (0.33)	0.03 (0.07)	197	**<0.0001**	0.13 (0.10)	0.02 (0.05)	186.5	**<0.0001**
Food area	Shelves area	0.48 (0.68)	0.17 (0.28)	158	**<0.0001**	–			
Food area×Gaze Food	Shelves area×Gaze Shelves	0.13 (0.33)	0 (0.03)	143	**<0.0001**	–			
GA owner/food	GA owner/shelves	–				3 (4)	0.5 (1)	143	**<0.0001**
Food area×GA owner/food	Shelves area×GA owner/shelves	–				1 (3)	0 (0)	89.5	**0.0003**
***Exit door (Food+Owner) vs. Exit door (Absence of Food)***									
Gaze Exit door	Gaze Exit Door	0.09 (0.16)	0.21 (0.35)	−124	**0.003**	0.07 (0.10)	0.07 (0.10)	−94.5	**0.028**
Exit door area	Exit door area	0 (0)	0 (0.11)	−22.5	**0.019**	–			
Exit door area×Gaze Exit door	Exit door area×Gaze Exit door	0 (0)	0 (0.04)	−22.5	**0.004**	–			
GA owner/exit door	GA owner/exit door	–				0 (1)	1 (1)	−87	**0.0006**
***Food (Food+Owner) vs. Exit door (Absence of Food)***									
Gaze Food	Gaze Exit door	0.26 (0.33)	0.21 (0.35)	5.5	0.908	0.13 (0.10)	0.07 (0.10)	94	**0.014**
Food area	Exit door area	0.14 (0.20)*	0 (0.08)*	103	**0.010**	–			
Food area×Gaze Food	Exit door area×Gaze Exit door	0.04 (0.10)*	0 (0.03)*	34.5	0.273	–			
GA owner/food	GA owner/exit door	–				3 (4)	1 (1)	116	**0.003**
Food area×GA owner/food	Exit door area×GA owner/exit door	–				0.29 (0.87)*	0 (0)*	68	**<0.0001**

GA: Gaze Alternation.

*corrected regarding relative size of the areas.

Obs: Gaze Alternation was only measured in absolute frequency, and variables that involved areas were only measured in duration.

Comparisons between these two conditions regarding behaviors towards the exit door intended to evaluate if the dogs showed the same interest in it (see statistics in Table 2). The time spent in Exit door area during Absence of Food was greater than during Food+Owner. The duration and frequency of Gaze Exit door and the frequency of Gaze Alternations between the owner and the exit door during Absence of Food was significantly greater than during Food+Owner. When Gaze Exit door was analyzed only in Exit door area, it was also found greater during Absence of Food. Gaze Alternations between the owner and the exit door specifically in Exit door area did not occur, and, therefore, could not be analyzed. In sum, dogs tended to show more interest in the exit door during the Absence of Food condition.

Comparisons of behaviors towards the food during the Food+Owner condition with behaviors towards the exit door during the Absence of Food condition intended to evaluate if the dogs communicated about the food when it was taken away to the same extent as when it was present in the room (see statistics in Table 2). The dogs spent less time in Exit door area during Absence of Food than in Food area during Food+Owner. However, the duration of Gaze Exit door during Absence of Food did not differ from the duration of Gaze Food during Food+Owner (Figure 6), even when analyzed in respective areas (duration). On the other hand, the frequency of Gaze Alternations between the owner and the food during Food+Owner was significantly greater than between the owner and the exit door during Absence of Food (Figure 7), even when analyzed in respective areas. In sum, whilst gazes at the food tended to divert to the exit door in the Absence of Food condition, gaze alternations were preferentially used when the food was present and accessible for the owner.

#### Test of the owner’s direction of attention (criterion d): Comparison between the Food+Owner and Owner Turned conditions

Comparisons between these two conditions regarding Vocalization and Sonorous Mouth Licking intended to evaluate if the acoustic behavior could have been increased as a way to get the owners’ attention during Owner Turned condition. These acoustic behaviors did not differ between Food+Owner and Owner Turned in durations (Vocalizations: T = −1.5, p = 0.813; Sonorous Mouth licking: T = −1, p = 0.953) and frequencies (Vocalizations: T = 2.5, p = 0.625; Sonorous Mouth licking: T = −2.5, p = 1.000), even when analyzed only in Food area (duration) (Vocalizations: T = 0, p = 1.000; Sonorous Mouth licking: T = −5, p = 0.125).

Likewise the duration (T = 30, p = 0.526) and frequency (T = 63.5, p = 0.151) of Gaze Food also did not differ between these conditions, even when analyzed only in Food area (duration: T = −6.5, p = 0.873). On the contrary, the duration (T = 175.5, p<0.0001) and frequency (T = 177, p<0.0001) of Gaze Owner (face) and the frequency of Gaze Alternations between the owner (face) and the food (T = 166, p<0.0001) were significantly greater during Food+Owner than during Owner Turned (when the dogs had to walk around the owner to see his/her face).

**Figure 8:**
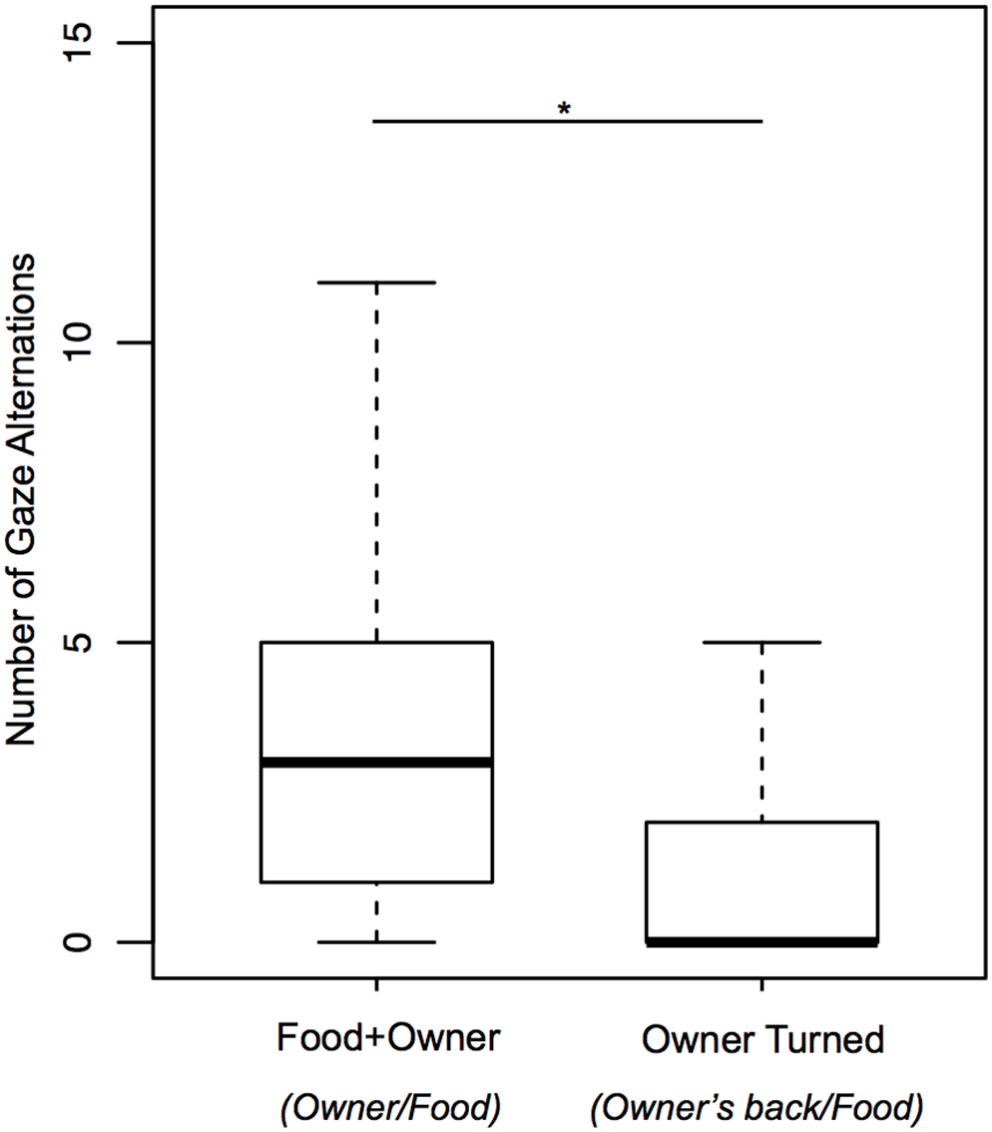
Frequencies of gaze alternation between the owner and the food during Food+Owner and between the owner’s back and the food during Owner Turned.

Comparisons of behaviors towards the owner’s face during the Food+Owner condition with behaviors towards the owner’s back during the Owner Turned condition intended to evaluate if the dogs tended to communicate more when their owners were facing the food than when they were not (see statistics Table 3). The duration and frequency of Gaze Owner (face) and the frequency of Gaze Alternations between the owner (face) and the food during Food+Owner were significantly greater than those towards the owner’s back during Owner Turned (Figure 8). However, when analyzed only in Food area (duration) no differences were found.

**Table 3:** Medians (Interquartile ranges-IQR) for the variables and two-sample Wilcoxon Signed-rank tests for comparisons regarding the effect of the owner’s direction of attention.

Comparisons		Duration: Median (IQR)				Frequency: Median (IQR)			
Food+Owner variable	Owner Turned variable	Food+Owner	Owner Turned	*T*	*p*	Food+Owner	Owner Turned	*T*	*p*
***Owner’s face (Food+Owner) vs. Owner’s Back (Owner Turned)***									
Gaze Owner	Gaze Owner’s back	0.24 (0.21)	0.11 (0.16)	153.5	**0.0003**	0.13 (0.10)	0.07 (0.10)	175.5	**<0.0001**
Food area×Gaze Owner	Food area×Gaze Owner’s back	0.08 (0.18)	0.03 (0.11)	74.5	0.090	–			
GA owner/food	GA owner’s back/food	–				3 (4)	0 (2)	99	**0.005**
Food area×GA owner/food	Food area×GA owner’s back/food	–				1 (3)	0 (1)	40	0.141
***Behaviors in Food area (Food+Owner) vs. in Back area (Owner Turned)***									
Food area×Gaze Owner	Back area×Gaze Owner	0.04 (0.09)*	0 (0.02)*	89	**0.029**	–			
Food area×Gaze Food	Back area×Gaze Food	0.06 (0.18)*	0 (0)*	136	**<0.0001**	–			
Food area×GA owner/food	Back area×GA owner/food	–				0.47 (1.41)*	0 (0)*	78.5	**0.0001**
Food area×Vocalization	Back area×Vocalization	0 (0)*	0 (0)*	−5	0.125	–			
Food area×Sonorous ML	Back area×Sonorous ML	0 (0)*	0 (0)*	2.5	0.625	–			

GA: Gaze Alternation.

ML: Mouth Licking.

*Corrected regarding relative size of the areas.

Obs: Gaze Alternation was only measured in absolute frequency, and variables that involved areas were only measured in duration.

Significant differences are in bold.

Comparisons of behaviors in Food area (duration) during the Food+Owner condition with behaviors in Back area during the Owner Turned condition intended to evaluate if the dogs modulated their behaviors according to the position in the room. The dogs spent more time in Back area during Owner Turned than during Food+Owner (T = −76, p = 0.017), however the time spent in Food area did not differ between these two conditions (T = 45, p = 0.339).

The duration of Gaze Owner, Gaze Food and Gaze Alternation between the owner and food in Food area during Food+Owner were greater than in Back area during Owner Turned (see statistics in Table 3). However, the duration of Vocalization and Sonorous Mouth Licking in Food area during Food+Owner did not differ from the duration of these behaviors in Back area during Owner Turned. Since these acoustic behaviors rarely occurred in Food area during Food+Owner, these results indicate that they also rarely occurred in Back area.

#### Test of persistence and elaboration (criteria e and f): Comparisons between the pre and post-delivery phases of Food, Half-Food and Undesirable Food conditions

These comparisons intended to evaluate the persistence of communicative behaviors or location (or combination of them) facing a complete or partial failure in communication. The duration and frequency of Gaze Owner, the time spent in Food area and the combination of Food area with Gaze Owner (duration) did not differ across phases and conditions after correcting for multiple comparisons (see statistics in Table S4).

On the other hand, the duration and frequency of Gaze Food decreased significantly from pre to post-delivery phases for both Food and Half-Food, but it did not decrease significantly for Undesirable Food, even when analyzed only in Food area (duration) (see statistics in Table 4). The comparisons between the post-delivery phases revealed no difference between Food and Half-Food (duration: T = −17.5, p = 0.698, frequency: T = 78.5, P = 0.057), even when analyzed only in Food area (duration: T = 2.5, p = 0.948). However, the duration of Gaze Food was significantly longer during the post-delivery phase of Undesirable Food than during the post-delivery phase of Food (duration: T = 101.5, p = 0.025), even when analyzed only in Food area (duration: T = 111, p = 0.003), whereas the frequency of Gaze Food did not differ significantly after correcting for multiple comparisons (T = 80, p = 0.039).

**Table 4:** Medians (Interquartile ranges-IQR) for the variables and two-sample Wilcoxon Signed-rank tests for comparisons regarding persistence.

	**Gaze Food**						**Gaze Alternation owner/food**		
	**Duration: Median (IQR)**			**Frequency: Median (IQR)**			**Frequency: Median (IQR)**		
**Condition**	**Pre**	**Post**	**T (p)**	**Pre**	**Post**	**T (p)**	**Pre**	**Post**	**T (p)**
***All of the room***									
Food	0.29 (0.36)	0.10 (0.21)	157.5 (***p<0.001***)	0.17 (0.16)	0.07 (0.10)	155.5 (***p<0.0001***)	3 (4)	2 (2)	107 (***p = 0.004***)
Half-Food	0.26 (0.34)	0.10 (0.15)	130 (***p<0.001***)	0.17 (0.20)	0.10 (0.10)	115.5 (***p = 0.003***)	4 (5)	3 (4)	21.5 (*p = *0.592)
Undesirable Food	0.20 (0.36)	0.16 (0.26)	59 (*p = *0.184)	0.10 (0.16)	0.10 (0.10)	23.5 (*p = *0.513)	2 (4)	2 (3)	20 (*p = *0.551)
***In Food Area***									
Food	0.17 (0.42)	0.03 (0.15)	118 (***p<0.001***)	–			1 (4)	1 (2)	62 (**p = 0.018**)
Half-Food	0.15 (0.35)	0.05 (0.16)	86 (***p = 0.011***)	–			1 (4)	1 (4)	−4 (p = 0.982)
Undesirable Food	0.14 (0.24)	0.14 (0.27)	5 (p = 0.902)	–			1 (3)	1 (3)	4 (p = 0.891)

Significant differences are in bold. *After the FDR BL adjustment, only the p-values shown in italics remain statistically significant.*

**Figure 9:**
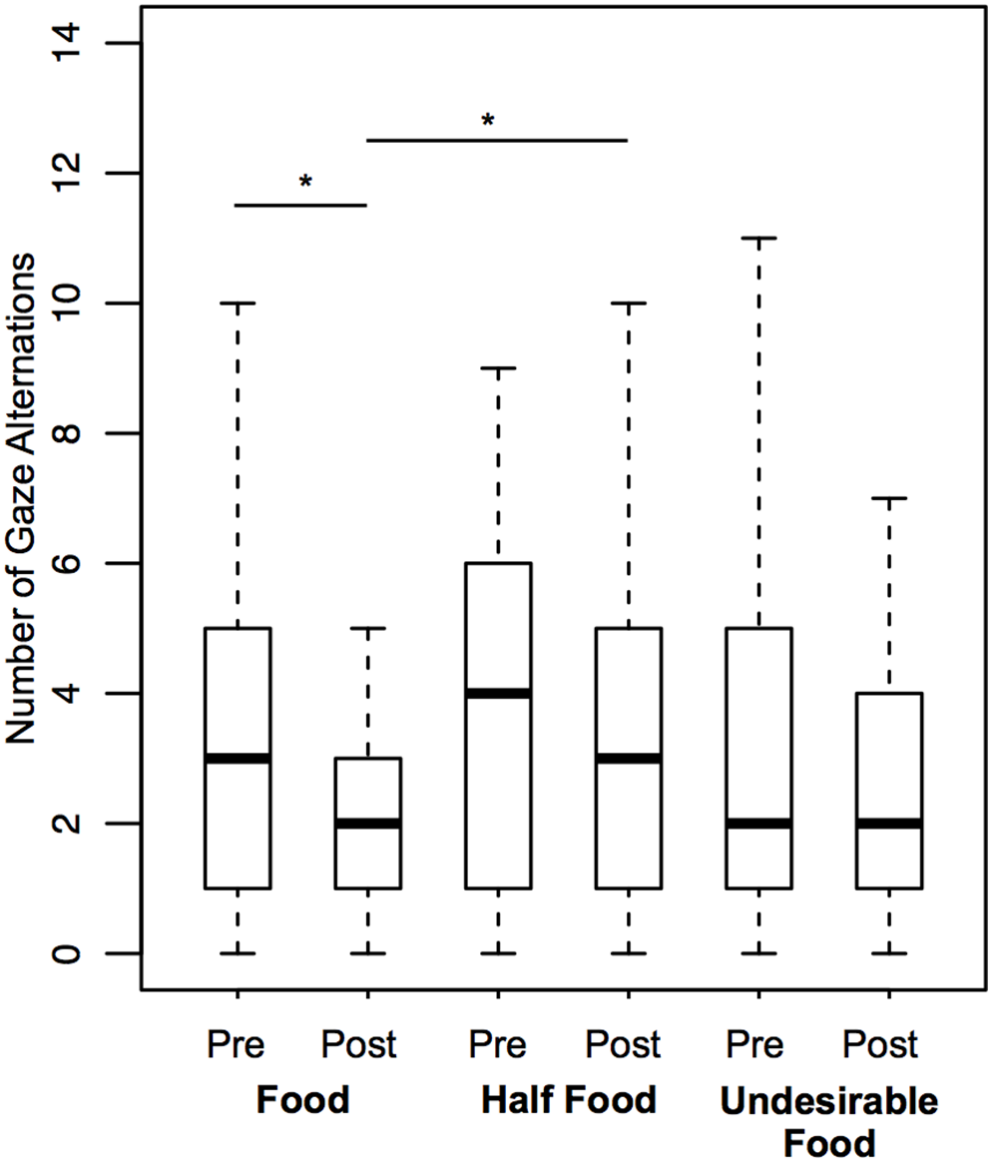
Frequencies of Gaze Alternation between the owner and the food in the pre and post-delivery phases for Food, Half-Food and Undesirable Food conditions.

The Gaze Alternation between the owner and the food significantly decreased from pre to post-delivery phase for Food while such difference was not found for Half-Food and Undesirable Food (see statistics in Table 4 and see Figure 9). This behavior was significantly greater during the post-delivery phase of Half-Food than during the post-delivery phase of Food (T = 110.5, p = 0.009), whereas there was no difference between the post-delivery phases of Food and Undesirable Food (T = 62.5, p = 0.110). When analyzed only in Food area the frequency of Gaze Alternation between the owner and the food presented the same tendency, but it did not differ significantly across phases and conditions.

The duration and frequency of Sonorous Mouth Licking did not differ between pre and post-delivery phases for the three conditions, as well as for the comparisons of the post-delivery phase of Food with Half-Food (see statistics in Table S4), which means that the dogs did not persist with this acoustic behavior.

**Figure 10:**
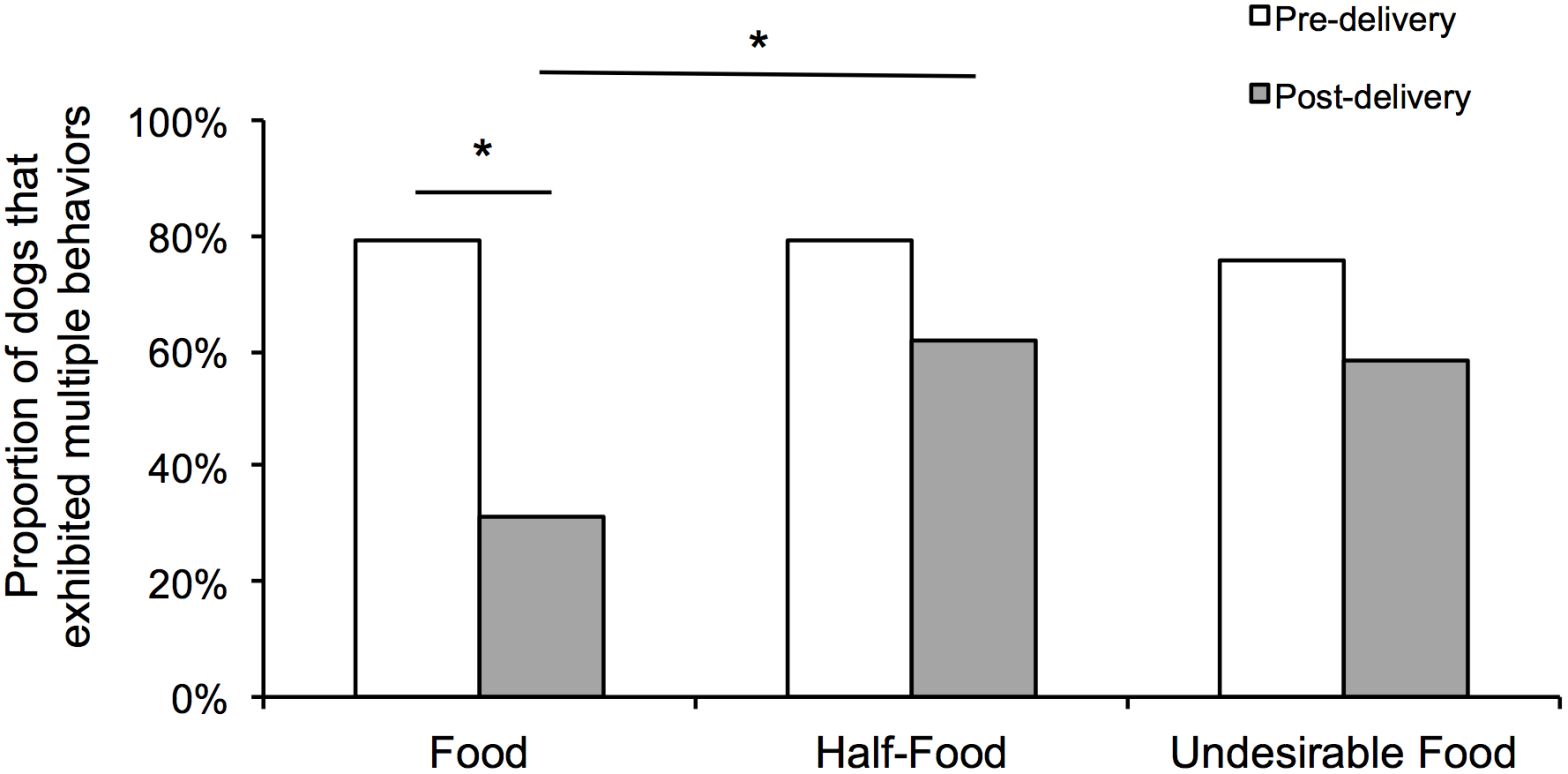
Proportion of dogs that exhibited multiple behaviors in each phase of Food, Half-Food and Undesirable Food conditions.

The comparisons among these three conditions also intended to address if the dogs used an elaborated repertoire of communicative behaviors when facing a complete or partial failure in communication. The proportion of dogs that exhibited multiple behaviors did not differ between the three pre-delivery phases (χ^2^ = 0.20, *df* = 2, p = 0.905; Figure 10). The proportion of dogs that exhibited multiple behaviors decreased significantly from pre to post-delivery phase for Food, while such decrease was not found for Half-Food and Undesirable Food (see statistics in Table 5). In the post-delivery phases, significantly more dogs exhibited multiple behaviors after the delivery of half of the food when compared to the entire food (χ^2^ = 7.36, *df* = 1, p = 0.007), but there was just a tendency of difference after the delivery of the undesirable food when compared to the entire food, which was not significant after correcting for multiple comparisons (χ ^2^ = 6.40, *df* = 1, p = 0.011). The proportion of dogs that exhibited multiple behaviors only in Food area did not differ between the three pre-delivery phases (χ^2^ = 0.12, *df* = 2, p = 0.943), as well as for the other comparisons across phases and conditions after correcting for multiple comparisons (see statistics in Table 5).

**Table 5:** Percentages of dogs that exhibited multiple behavior (MB) with Mc Nemar’s tests, and, absolute frequency of MB with two-sample Wilcoxon Signed-rank tests for the comparisons regarding elaboration.

Variable	Condition	*Phase*		*Statistics (p)*
**% of dogs that exhibited MB**				
	***All of the room***	**Pre**	**Post**	?**^2^** ***df*** ** = 1 (p)**
	Food	79.3%	31.0%	*14 (* ***p<0.001*** *)*
	Half-Food	79.3%	62.1%	2.27 (*p = *0.132)
	Undesirable Food	75.9%	58.6%	2.27 (*p = *0.132)
	***In Food area***			
	Food	55.2%	27.6%	5.33 (**p = 0.021**)
	Half-Food	55.2%	41.4%	1.60 (*p = *0.206)
	Undesirable Food	58.6%	44.8%	1.60 (*p = *0.206)
**Frequency of MB: Median (IQR)**				
	***All of the room***	**Pre**	**Post**	**T (p)**
	Food	1 (2)	0 (1)	−89 ***(p<0.001)***
	Half-Food	1 (2)	1 (2)	−45.5 (*p = *0.136)
	Undesirable Food	1 (2)	1 (3)	−18 (*p = *0.450)
	***In Food area***			
	Food	1 (2)	0 (1)	−45 (**p = 0.011**)
	Half-Food	1 (2)	0 (1)	−27.5 (p = 0.123)
	Undesirable Food	1 (2)	0 (2)	−13 (*p = *0.552)

MB: Multiple Behaviors.

Significant differences are in bold. *After the FDR BL adjustment, only the p-values shown in italics remain statistically significant.*

The frequency of multiple behaviors did not differ between the three pre-delivery phases (χ^2^ = 0.06, *df* = 2, p = 0.808). It decreased significantly from pre to post-delivery phase for Food, while no such decrease was found for Half-Food and Undesirable Food (see statistics in Table 5). In the post-delivery phases of Food and Half-Food no difference was found (T = 46.5, p = 0.039) after correcting for multiple comparisons, however significantly more multiple behaviors were exhibited after the delivery the undesirable food (T = 48, p = 0.004) when compared to the entire food. The frequency of multiple behaviors displayed only in Food area did not differ between the three pre-delivery phases (χ^2^ = 0.22, *df* = 2, p = 0.896), as well as for the other comparisons across phases and conditions after correcting for multiple comparisons (see statistics in Table 5).

Finally, the proportion of dogs that exhibited alternative behaviors during the post-delivery phase, not displayed during the pre-delivery phase, did not significantly differ between Food (31%), Half-Food (44.8%) and Undesirable Food (31%) (χ^2^ = 1.9, *df* = 2, p = 0.390). When analyzed only in Food area the proportion of dogs that exhibited alternative behaviors was 20.7% for all three conditions (χ^2^ = 0, *df* = 2, p = 1.000).

## Discussion

The operational criteria of referentiality and intentionality have been investigated in different studies with dogs. Miklósi and colleagues [5] and Gaunet and Deputte [6] addressed criteria a-c and found that gazes and gaze alternations are influenced by the audience and are used in a functionally referential way. Gaunet and Deputte [6] also suggested that dogs use their position as a local enhancement cue. The direction of human’s attention influenced dogs’ gaze alternations when they faced a potentially scary object or an unsolvable task [37], [38] (criterion d). Finally, one study [7] suggested that dogs persist (criterion e) when they request a toy and receive an unfamiliar object instead, however this study [7] challenged the elaboration of communication (criterion f). The current study integrated all these hypotheses and addressed at the same time all operational criteria. The choice of a visible food allowed us to concentrate on the communication without any interference from memory that a hidden target could yield. Additionally, by using food that could be divided into two, it was possible to extend the results regarding persistence and elaboration proposed by Gaunet [7] by introducing a situation of partial failure in communication.

Our results converge with previous studies [5], [6] confirming that the behaviors gaze at the owner, gaze at the food, gaze alternation between the owner and the food, the sonorous mouth licking and the use of the food area (either accompanied or unaccompanied by gaze at the owner, gaze at the food and gaze alternations) are socially used, i.e. meet the criterion of communication and audience effect (criterion a).

The dogs gazed less at the food when their owners were absent than when they were present, however there was no such effect when this behavior was analyzed specifically in the food area, which suggests that in the presence of the owner gazing at the food does not only occur close to the food, but instead it is also a distal signal that can be displayed from other places in the room.

Regarding criteria b and c (related to referentiality) our results suggest that the dogs gazed longer at the owners when the food was present than when it was absent, and this effect was also observed when this behavior was analyzed specifically when the dogs were close to the food/shelves areas. This shows that the dogs referred visually to the owners as if trying to get their attention or waiting for/seeking a response. In Gaunet and Deputte [6], gaze at the owner did not differ between similar conditions (presence or absence of a toy); however, in that study the owner was both the hider of the toy and the provider, which could have led the dogs to maintain their gaze at the owner even in the absence of the toy in order to continue the interaction. In the current study, the fact that these two roles were played by different people allowed the referentiality of gazing at the owner to be emphasized. Additionally, this behavior combined with positioning close to the food, suggests that the dogs used their own body as a local enhancement cue (gazes at the owner in the food area happened more than in the shelves area when there was no food) as if they were taking the visual perspective of their owners. This is in agreement with previous studies [6], [35], [36]. A parsimonious interpretation can, however, also be given: dogs could have learned that gazing at the owner next to the desirable food increases the odds of being rewarded.

We also observed that the dogs positioned themselves next to the food shelf for a longer period of time than next to the shelves in the absence of the food. Moreover, the gazes at the food and the gaze alternation between the owner and the food in the presence of both were also significantly longer and more frequent than those towards the shelves when the food was absent (this also occurred when the dogs were specifically in the food/shelves area, respectively), showing the functionally referential properties of these behaviors also when combined with being in the food area.

The analyses regarding the exit door also revealed important evidence concerning referentiality since it represents the direction of the food’s location when the helper took it away in the Absence of Food condition. The dogs spent more time next to the exit door, gazed more at the exit door (whether they were located in the exit door area or not) and alternated more gazes between the owner and the exit door when the helper took the food away than when the food was present. In fact, regarding duration, the dogs gazed at the exit door when the food was absent at the same rate as they did towards the food when it was present, (this also occurred when dogs were specifically in the exit door/food area, respectively), which suggests that gazes at the food, and this behavior combined with location, diverted towards the exit door when the helper took the food away. On the other hand, the gaze alternations did not divert towards the exit door when the food was taken away, and, the dogs still spent more time next to the food when it was present than next to the exit door when the food was absent.

Similar to the findings of Gaunet and Deputte [6], gaze at the food, gaze alternation and the use of position in the room do not have the same function when the target is present or absent. Gazes at the exit door, the time spent next to this door and the combination of them when the helper took the food away may be a “waiting” reaction, while gaze alternation may be used to request the food when it is present. The dogs acted as if it was less “worthwhile” requesting the food by alternating gazes when it was not accessible for the owner, or, as if they had previously learnt that requesting an out-of-reach food does not lead to a positive response.

Regarding the sonorous mouth licks, even though we previously found this to be a communicative behavior, it was not used referentially in the present study: there was no effect of the absence of the food on this behavior. Gaunet and Deputte’s [6] results showed no effect of the absence of the owner or toy on sonorous mouth licks and they suggested that this could be explained by the fact that the target was a toy instead of food. However, by using food as a target, we found that the audience influences this behavior, but it is not referential.

With respect to the criterion d (effect of the direction of the owner’s attention), the dogs gazed more frequently and longer at the owners’ face and alternated more gazes towards the owners’ face when they were facing the experimental setting than towards the owners’ back when they had their back turned, in agreement with the study that evaluated this effect in an unsolvable task [38]. This suggests that gazing at the owner’s face is an attention-seeking behavior rather than a checking behavior. Although the tendency was the same when this analysis was repeated while dogs were only in the food area (see descriptive measures in Table 3), the difference was not significant. We can infer that gazing towards the owners and gaze alternations when they were facing the experimental setting also happened outside the food area, an indication that the direction of attention influenced these behaviors, but it did not influence the use of the location in order to enhance them.

Actually, the owner’s direction of attention did not significantly affect the time spent in the food area. However, it is important to notice that the time spent in the back area increased when owners had their back turned, which suggests that dogs tended to adjust their own position to be face-to-face with owners.

We also found that gazing at the food and its combination with the food area did not differ when owners were facing the experimental setting or had their back turned, probably because the dogs were still attracted by the presence of the food.

This study, therefore, provides us with some evidence that dogs use the owner’s body orientation to modulate some behaviors. The dogs also tended to adjust their position by moving around the owners to face them in the Owner Turned condition, as chimpanzees did in the study of Liebal, Call and Tomasello [45]. Nevertheless, after moving around, the dogs did not gaze at the owner’s face, at the food or alternate gazes between them as a way of getting the owner’s attention as much as they did when the owners were facing towards the food. A possible explanation is that dogs seem to take into account what humans can or cannot see and do not display directional behaviors towards an object in the environment that is not in the owners’ visual field. Such explanation is supported by findings in previous studies [33], [35], [36]. However, dogs could also have learned that communicative behaviors towards the food, when owners are not facing it, do not result in the provision of food.

Finally there was no effect of the direction of the owners’ bodies on sonorous mouth licks and vocalizations. A possible explanation for the absence of vocalizations also in the Owner Turned condition could be that owners usually discourage dogs from barking and they could have learned that this behavior should be avoided regardless the context. These results are in agreement with Gaunet [7] who also found that dogs did not use acoustic behaviors to get the owner’s attention.

Altogether the owner’s body direction influenced the use of gaze alternations between the owner and the food and gazes at the owner, which seem to be attention-seeking behavior rather than a checking/anticipatory behavior. A high-level interpretation would suggest an ability that implies “understanding” of human attentional state, however a parsimonious interpretation can be given since dogs may learn about the implications of human body orientation in communicative interactions in their daily life experiences.

We also found evidence of persistence (criterion e) for the two communicative behaviors directed towards the food, gazing at the food (in agreement with Gaunet [7]) and gaze alternation between the owner and the food. While the duration and frequency of gazes at the food decreased significantly after receiving the entire food, it did not decrease after receiving the undesirable food, which shows persistence in this situation. The comparisons in the post-delivery phases revealed that the dogs gazed significantly longer at the food when the attempt to communicate completely failed than when it was successful, which confirms the persistence of gazing at the food when faced with the complete failure in communication. Conversely, there was a significant decrease in the duration and frequency of gazes at the food after receiving half of the food. This means that the dogs did not persist in gazing at the food when they received half of it. When gazing at the food was analyzed specifically in the food area, we observed the same pattern. Therefore, the dogs used to gaze at the food and also this behavior combined with their own body position as an enhancement cue to persist after receiving the undesirable food.

A different pattern was observed regarding gaze alternation between the owner and the food. The results showed that while the frequency of gaze alternations significantly decreased after receiving the entire food, there was no such decrease after receiving half of the food and the undesirable food, evidence for persistence facing these two outcomes of communication. The comparisons in the post-delivery phases confirmed the persistence only for the partial failure of communication: the dogs alternated significantly more gazes between the owner and the remaining food after receiving half of the food than between the owner and the empty shelf after receiving the entire food. No difference was found after receiving the undesirable food and the entire food; therefore, the persistence with gaze alternations when facing the complete failure in communication (no difference between pre and post-delivery phase for undesirable food condition) should be considered as a tendency. When the frequency of gaze alternations was analyzed specifically in the food area, no differences were found across phases and conditions, which implies that the persistence observed for this behavior did not happen specifically in the food area, but across the whole room.

Regarding total failure of communication, we found a different tendency compared to the previous study [7]. Gaunet found a decrease in the frequency of gaze alternations after returning an unfamiliar object to the dog (analogous to our undesirable food condition). This was attributed to the nature of the target, after receiving the unfamiliar object dogs spent some time sniffing it and consequently there was no time left to other behaviors like gaze alternation, which suggests a distinct differential values between desirable and undesirable food vs. toy and new object. Since this previous study [7] could not evaluate the partial failure situation, the current research brings new information about the function of gaze alternation: it is indeed a referential communicative behavior that persists when the recipient of a message appears to be available to cooperate by giving part of the food requested.

Altogether, results observed for dogs, in a situation where they could beg for food, were similar to findings for chimpanzees [12]. The dogs used different strategies to persist depending on the outcome of the communication. This could potentially be explained by experience acquired during their lives: for undesirable food, dogs showed persistence for both behaviors, gazes at the food and gaze alternations – even though it was less clear for gaze alternations – as if, in the past, they had learned that their owners were “less cooperative” in such situation. On the other hand, for the Half-Food condition, a different strategy was at play (i.e., only gaze alternations were maintained), which may have been learned as sufficient to continue manipulating the owners when they had partially answered the request and “were willing to cooperate” by giving a piece of food. Jointly, these results confirm not only persistence but also an ability to discriminate between being given food (whether this is the entire amount or only half of it) or not.

It is important to notice that gaze at the owner (whether dogs were located in the food area or not) was not used by the dogs to persist when the communication failed or partially failed. Actually, there was no decrease in this behavior even after they had received the entire food. A plausible explanation is that dogs continue expecting or soliciting interaction with their owners even after eating the food.

The sonorous mouth licks were not displayed in order to persist when the communication failed. It is important to emphasize that it was necessary to consider an adjustment in the persistence and elaboration analyses in order to prevent the inclusion of mouth licks that occurred just after eating food, which could be an immediate mouth cleaning reaction. Since this behavior can have a mixed interpretation in food begging situation, caution is needed when analyzing this result. New studies with other approaches are required in order to have a better understanding of this behavior in this context.

Regarding the *elaboration* (criterion f), we observed that, while after receiving the entire food the multiple behaviors decreased significantly (proportion of dogs that used them and their frequency per se), after receiving half of the food or an undesirable food, the dogs continued presenting an elaborate behavioral repertoire. In the post-delivery phase, the proportion of dogs that exhibited multiple behaviors after receiving half of the food was significantly greater than after receiving the entire food, while no significant difference was found after receiving an undesirable food compared with the entire food. Conversely, in the post-delivery phase the frequency of multiple behaviors when the communication completely failed was greater than when it was successful, while no such difference was found between the partial and successful communication. Therefore, each approach brought evidence of elaboration in one of the outcomes of communication.

A different result was observed for the proportion of dogs that exhibited alternative behaviors in post-delivery phases, no difference across conditions was observed, which is in agreement with Gaunet [7], who found no new communicative behaviors after the communication had failed. The lack of alternative (or new) behaviors after the communicative failure could indicate that dogs do not elaborate the communication, however, alternatively, this result could also indicate that dogs might use their entire repertoire of behaviors from the outset in order to achieve their goals; therefore, possibly this measure is not capable of providing evidence of the elaboration of communication.

Leavens and colleagues [12] found that the possession of a half-banana or an undesirable food did not suppress multiple gestures in chimpanzees, and they advocated that this result indicates elaboration for both outcomes. However, in the post-delivery phase significantly more chimpanzees exhibited multiple gestures after the complete failure in communication than after its success, and no such difference was found between partial failure and success. This is a different behavior pattern when compared to dogs, since we found stronger evidences that more dogs tended to use an elaborate behavioral repertoire when facing partial failure than when facing the complete failure. This tendency could also be a result of experience: the dogs can have learnt that their owners are more willing to give more food when they had already given a part of it than when they had given something that was not interested for the dogs.

It was observed that gazes at the food were not influenced by the direction of the owner’s body, and, gazes at the owner were not diferentially displayed when the communication succeeded or failed; therefore, these two behaviors failed to meet some criteria of referentiality and intentionality. Nevertheless, it should be considered that gazes at the owner and at the food are directly connected to the production of gaze alternations between them. The gaze alternation between the owner and the food, is, in fact, the central behavior of referential and intentional communication and it was widely used by the dogs in the current study (criterion a). It is a referential communicative behavior (criteria b–c), i.e. it refers, in fact, to the food, and, it is also influenced by the direction of the owner’s attention (criterion d). Finally, the dogs persisted with gaze alternation when the attempt to manipulate their owners partially failed (criterion e), and there was a tendency to persist after the communication completely failed. We propose that even though gaze at the owner and at the food failed in some criteria, this did not invalidate the attribution of referentiality and intentionality for the dogs’ communication, since the results for the gaze alternations were convincing.

The elaboration of communication (criterion f) is still the most challenging criterion to be validated due to the difficulty to assess it. By using a similar definition suggested by Leavens and colleagues [12] we found evidence that this criterion is also met for dogs.

Owners usually claim that their dogs use gaze and their own position to indicate desires, such as the place where the food is stored or the location of the leash they wear when they go for a walk. Overall, not all criteria were met for the gaze at the owner and gaze at the food, as well as, for vocalization, mouth lickings, and for the use of the position in the room. On the other hand, we found strong evidences that the gaze alternation between the owner and the food met criteria a–e and, additionally, the dogs continued using an elaborated repertoire of behaviors when the communication partially failed, which jointly suggests that dogs are able to communicate in a functionally referential and intentional way. However, this does not mean they have a theory of mind about their owner’s motives or that this could reflect an “understanding” of their owner’s mental state. We cannot exclude that learning plays a role in the development of communicative behaviors in dogs. Bentosela and colleagues [46] showed that even the gaze response can be quickly learned by dogs. Nevertheless, the incidental learning in the experimental procedure used in our study would not be a sufficient explanation for the dogs’ observed behavior.

Since a large number of tests was necessary in order to evaluate all criteria at the same time, the possibility of inflating type I error should not be excluded, even performing corrections for multiple tests when necessary. Nevertheless, the current study brings to light the complexity of dog’s communicative behavior. It is plausible to use an “*evo-devo*” approach to explain dog’s behavior. Undoubtedly the learning and experiences during lifetime have an important role in shaping the communication established by the dyad owner and dog, but it is not possible to exclude a “predisposition” to communicate in this special manner [5]. From an evolutionary perspective, communicating with humans and being especially able to be attuned to cues relating to their attention (i.e., to adjust to them) in order to request food in an apparent referential and intentional way may have brought a selective advantage for dogs.

## Supporting Information

Table S1 **Medians (Interquartile ranges-IQR) for the variables and two-sample Wilcoxon Signed-rank test for comparisons of the first and last pre-delivery phases (from Food, Half Food or Undesirable Food conditions).**
(DOCX)

Table S2 **Medians (Interquartile ranges-IQR) for the variables, and Friedman tests for comparisons of the three pre-delivery phases of Food, Half-food and Undesirable (Und.) Food conditions (** ***df*** **= 2).**
(DOCX)

Table S3 **Medians (Interquartile ranges-IQR) for the variables and one-sample Wilcoxon Signed-rank tests to evaluate if the variables differ from zero in the pre-delivery phase with Food+Owner (F+O) condition**. Significant differences are in bold.
(DOCX)

Table S4 **Medians (Interquartile ranges-IQR) for some variables and two-sample Wilcoxon Signed-rank tests for comparisons regarding persistence.** Significant differences are in bold. After the FDR BL adjustment, only the p-values shown in italics remain statistically significant.
(DOCX)

Video S1 **Bartolomeu and Paulo in Food condition.** The video illustrates the pre-delivery and post-delivery phases of Food condition.
(MOV)

Video S2 **Bartolomeu and Paulo in Half-Food condition.** The video illustrates the pre-delivery and post-delivery phases of Half-Food condition.
(MOV)
