# Correction: Are Dogs Able to Communicate with Their Owners about a Desirable Food in a Referential and Intentional Way?

**DOI:** 10.1371/journal.pone.0111387

**Published:** 2014-10-13

**Authors:** The PLOS ONE Staff

## Notice of Republication

This article was republished on September 30, 2014, to correct an error in the third author’s affiliation and in the Author Contributions section that were introduced during the production process. The publisher apologizes for the errors. Please download this article again to view the correct version. The originally published, uncorrected article and the republished, corrected article are provided here for reference.

## Supporting Information

File S1 Originally published, uncorrected article.
(PDF)

File S2 Republished, corrected article.
(PDF)
