# Supplementary material for: Are Dogs Able to Communicate with Their Owners about a Desirable Food in a Referential and Intentional Way?
Source: PLoS One. 2014 Sep 18;9(9):e108003. doi: 10.1371/journal.pone.0108003 (PMC4169500; doi:10.1371/journal.pone.0108003)
Supplement: Table S1 — Medians (Interquartile ranges-IQR) for the variables and two-sample Wilcoxon Signed-rank test for comparisons of the first and last pre-delivery phases (from Food, Half Food or Undesirable Food conditions). (DOCX) [file pone.0108003.s001.docx]

**Table S1** Medians (Interquartile ranges-IQR) for the variables and two-sample Wilcoxon Signed-ranks test for comparisons of the first and last pre-delivery phases (from Food, Half Food or Undesirable Food conditions).

|  | **Median (IQR)** | | | |
| --- | --- | --- | --- | --- |
| **Variables** | **First pre-delivery phase** | **Last pre-delivery phase** | ***T*** | ***p*** |
| Gaze Owner duration | 0.22 (0.19) | 0.24 (0.23) | -52.5 | 0.264 |
| Gaze Food duration | 0.23 (0.37) | 0.21 (0.28) | -14.5 | 0.760 |
| GA owner/food | 2 (5) | 3 (5) | -30 | 0.339 |
| Food area duration | 0.45 (0.72) | 0.52 (0.63) | -32 | 0.452 |

GA – Gaze Alternation
