# Supplementary material for: Are Dogs Able to Communicate with Their Owners about a Desirable Food in a Referential and Intentional Way?
Source: PLoS One. 2014 Sep 18;9(9):e108003. doi: 10.1371/journal.pone.0108003 (PMC4169500; doi:10.1371/journal.pone.0108003)
Supplement: Table S2 — Medians (Interquartile ranges-IQR) for the variables, and Friedman tests for comparisons of the three pre-delivery phases of Food, Half-food and Undesirable (Und.) Food conditions ( df = 2). (DOCX) [file pone.0108003.s002.docx]

**Table S2** Medians (Interquartile ranges-IQR) for the variables, and Friedman tests for comparisons of the three pre-delivery phases of Food, Half-food and Undesirable (Und.) Food conditions (df=2).

|  | **Duration: Median (IQR)** | | | | | **Frequency: Median (IQR)** | | | | |
| --- | --- | --- | --- | --- | --- | --- | --- | --- | --- | --- |
| **Variables** | **Food** | **Half-food** | **Und. Food** | χ2 | ***p*** | **Food** | **Half-food** | **Und. Food** | χ2 | ***p*** |
| Gaze Owner | 0.22 (0.25) | 0.27 (0.30) | 0.24 (0.25) | 2.69 | 0.261 | 0.17 (0.13) | 0.17 (0.10) | 0.13 (0.10) | 0.40 | 0.820 |
| Gaze Food | 0.29 (0.36) | 0.26 (0.34) | 0.20 (0.36) | 2.48 | 0.289 | 0.17 (0.17) | 0.17 (0.20) | 0.10 (0.17) | 4.01 | 0.134 |
| GA owner/food | ⎯⎯⎯ | | | | | 3 (4) | 4 (5) | 2 (4) | 1.94 | 0.378 |
| Vocalization | 0 (0) | 0 (0) | 0 (0) | 1.35 | 0.508 | 0 (0) | 0 (0) | 0 (0) | 0.60 | 0.741 |
| ML | 0 (0) | 0 (0) | 0 (0) | 1.12 | 0.572 | 0 (0) | 0 (0) | 0 (0) | 1.23 | 0.542 |
| Sonorous ML | 0 (0) | 0 (0.04) | 0 (0) | 2.43 | 0.297 | 0 (0) | 0 (0.03) | 0 (0) | 2.98 | 0.226 |
| Contact Owner | 0 (0) | 0 (0) | 0 (0) | 2.82 | 0.244 | 0 (0) | 0 (0) | 0 (0) | 2.00 | 0.368 |
| Food area | 0.49 (0.82) | 0.49 (0.70) | 0.48 (0.69) | 0.13 | 0.937 | ⎯⎯⎯ | | | | |

GA – Gaze Alternation

ML – Mouth Licking
