# Supplementary material for: Are Dogs Able to Communicate with Their Owners about a Desirable Food in a Referential and Intentional Way?
Source: PLoS One. 2014 Sep 18;9(9):e108003. doi: 10.1371/journal.pone.0108003 (PMC4169500; doi:10.1371/journal.pone.0108003)
Supplement: Table S3 — Medians (Interquartile ranges-IQR) for the variables and one-sample Wilcoxon Signed-rank tests to evaluate if the variables differ from zero in the pre-delivery phase with Food+Owner (F+O) condition. Significant differences are in bold. (DOCX) [file pone.0108003.s003.docx]

**Table S3** Medians (Interquartile ranges-IQR) for the variables and one-sample Wilcoxon Signed-rank tests to evaluate whether the variables differ from zero in the pre-delivery phase with Food+Owner (F+O) condition. Significant differences are in bold.

|  | **Duration: Median (IQR)** | | | **Frequency: Median (IQR)** | | |
| --- | --- | --- | --- | --- | --- | --- |
| **Variables** | **F+O** | ***T*** | ***p*** | **F+O** | ***T*** | ***p*** |
| Gaze Owner | 0.24 (0.21) | 217.5 | **<0.0001** | 0.13 (0.10) | 217.5 | **<0.0001** |
| Gaze Food | 0.26 (0.33) | 217.5 | **<0.0001** | 0.13 (0.10) | 217.5 | **<0.0001** |
| GA owner/food | ⎯⎯⎯ | | | 3 (4) | 162.5 | **<0.0001** |
| Vocalization | 0 (0) | 5.0 | 0.125 | 0 (0) | 5.0 | 0.125 |
| ML | 0 (0) | 5.0 | 0.125 | 0 (0) | 5.0 | 0.125 |
| Sonorous ML | 0 (0) | 10.5 | **0.030** | 0 (0) | 10.5 | **0.030** |
| Contact Owner | 0 (0) | 0.5 | 1.000 | 0 (0) | 0.5 | 1.000 |
| Food Area | 0.48 (0.67) | 189 | **<0.0001** | ⎯⎯⎯ | | |
| Food area x Gaze Owner | 0.08 (0.18) | 150 | **<0.0001** | ⎯⎯⎯ | | |
| Food area x Gaze Food | 0.13 (0.33) | 126.5 | **<0.0001** | ⎯⎯⎯ | | |
| Food area x GA owner/food | 1 (3) | 68 | **<0.0001** | ⎯⎯⎯ | | |
| Food area x Vocalizations | 0 (0) | 3 | 0.250 | ⎯⎯⎯ | | |
| Food area x Sonorous ML | All zero ***-*** Not tested | | | ⎯⎯⎯ | | |

GA – Gaze Alternation

ML – Mouth Licking
