# Supplementary material for: Are Dogs Able to Communicate with Their Owners about a Desirable Food in a Referential and Intentional Way?
Source: PLoS One. 2014 Sep 18;9(9):e108003. doi: 10.1371/journal.pone.0108003 (PMC4169500; doi:10.1371/journal.pone.0108003)
Supplement: Table S4 — Medians (Interquartile ranges-IQR) for some variables and two-sample Wilcoxon Signed-rank tests for comparisons regarding persistence. Significant differences are in bold. After the FDR BL adjustment, only the p-values shown in italics remain statistically significant. (DOCX) [file pone.0108003.s004.docx]

**Table S4** Medians (Interquartile ranges-IQR) for some variables and two-sample Wilcoxon Signed-rank tests for comparisons regarding persistence. Significant differences are in bold. After the FDR BL adjustment, only the p-values shown in italics remain statistically significant.

|  |  | **Duration** | | | | **Number** | | | |
| --- | --- | --- | --- | --- | --- | --- | --- | --- | --- |
| **Variable** | **Condition** | **Median (IQR) Pre** | **Median (IQR) Post** | **Comparisons** | **T (p)** | **Median (IQR) Pre** | **Median (IQR) Post** | **Comparisons** | **T (p)** |
| Gaze Owner (B) | Food | 0.22 (0.25) | 0.19 (0.24) | Pre x Post | 12 (*p=*0.800) | 0.17 (0.13) | 0.10 (0.10) | Pre x Post | 84 (***p=*0.041**) |
|  | Half-Food | 0.27 (0.30) | 0.27 (0.37) | Pre x Post | *-*47.5 (*p=*0.313) | 0.17 (0.10) | 0.17 (0.07) | Pre x Post | -9 (*p=*0.802) |
|  | Und. Food | 0.24 (0.24) | 0.17 (0.32) | Pre x Post | 39 (*p=*0.409) | 0.13 (0.10) | 0.13 (0.07) | Pre x Post | 4.5 (*p=*0.916) |
|  |  |  |  | Post - Food x Half-Food | 105.5 (***p=*0.020**) |  |  | Post - Food x Half-Food | 92.5 (***p*=0.015**) |
|  |  |  |  | Post - Food x Und. Food | 12.5 (*p=*0.792) |  |  | Post - Food x Und. Food | 34.5 (*p=*0.391) |
| Sonorous ML (B) | Food | 0 (0) | 0 (0) | Pre x Post | −4.5 (*p=*0.717) | 0 (0) | 0 (0) | Pre x Post | −2.5 (*p=*0.841) |
|  | Half-Food | 0 (0.04) | 0 (0) | Pre x Post | 25.5 (*p=*0.045) | 0 (0.03) | 0 (0) | Pre x Post | 21 (*p=*0.073) |
|  | Und. Food | 0 (0) | 0 (0) | Pre x Post | 2 (*p=*0.844) | 0 (0) | 0 (0) | Pre x Post | -2 (*p=*1.000) |
|  |  |  |  | Post - Food x Half-Food | −11 (*p=*0.211) |  |  | Post - Food x Half-Food | −6.5 (*p=*0.328) |
|  |  |  |  | Post - Food x Und. Food | −15.5 (p=0.074) |  |  | Post - Food x Und. Food | −12.5 (p=0.188) |
| Food area (L) | Food | 0.49 (0.81) | 0.28 (0.46) | Pre x Post | 80.5 (p=0.082) | ⎯ | ⎯ | ⎯ | ⎯ |
|  | Half-Food | 0.49 (0.71) | 0.62 (0.68) | Pre x Post | -50.5 (p=0.222) | ⎯ | ⎯ | ⎯ | ⎯ |
|  | Und. Food | 0.48 (0.69) | 0.48 (0.68) | Pre x Post | 8 (p=0.842) | ⎯ | ⎯ | ⎯ | ⎯ |
|  |  |  |  | Post - Food x Half-Food | 68 (p=0.123) |  |  | ⎯ | ⎯ |
|  |  |  |  | Post - Food x Und. Food | 81 (p=0.050) |  |  | ⎯ | ⎯ |
| Food area x | Food | 0.06 (0.23) | 0.05 (0.11) | Pre x Post | 25 (p=0.558) | ⎯ | ⎯ | ⎯ | ⎯ |
| Gaze Owner | Half-Food | 0.08 (0.18) | 0.11 (0.34) | Pre x Post | -74 (**p=0.044**) | ⎯ | ⎯ | ⎯ | ⎯ |
| (LxB) | Und. Food | 0.09 (0.20) | 0.06 (0.13) | Pre x Post | -47 (p=0.267) | ⎯ | ⎯ | ⎯ | ⎯ |
|  |  |  |  | Post - Food x Half-Food | 86 (**p=0.036**) |  |  | ⎯ | ⎯ |
|  |  |  |  | Post - Food x Und. Food | 34.5 (p=0.364) |  |  | ⎯ | ⎯ |

B – Behavior

L – Location

LxB – Location x Behavior

GA – Gaze Alternation

ML – Mouth Licking
